# The statistical shape of geometric reasoning

**DOI:** 10.1038/s41598-018-30314-y

**Published:** 2018-08-27

**Authors:** Yuval Hart, Moira R. Dillon, Andrew Marantan, Anna L. Cardenas, Elizabeth Spelke, L. Mahadevan

**Affiliations:** 1 Paulson School of Engineering and Applied Sciences, Harvard University, Cambridge, MA 02138 USA; 2 Department of Psychology, New York University, New York, NY 10003 USA; 3 Department of Physics, Harvard University, Cambridge, MA 02138 USA; 4 Department of Psychology, Harvard University, Cambridge, MA 02138 USA; 5 Center for Brain Science, Harvard University, Cambridge, MA 02138 USA; 6 Department of Organismic and Evolutionary Biology, Harvard University, Cambridge, MA 02138 USA; 7 The Kavli Institute for Bionano Science and Technology, Harvard University, Cambridge, MA 02138 USA

## Abstract

Geometric reasoning has an inherent dissonance: its abstract axioms and propositions refer to perfect, idealized entities, whereas its use in the physical world relies on dynamic perception of objects. How do abstract Euclidean concepts, dynamics, and statistics come together to support our intuitive geometric reasoning? Here, we address this question using a simple geometric task – planar triangle completion. An analysis of the distribution of participants’ errors in localizing a fragmented triangle’s missing corner reveals scale-dependent deviations from a deterministic Euclidean representation of planar triangles. By considering the statistical physics of the process characterized via a correlated random walk with a natural length scale, we explain these results and further predict participants’ estimates of the missing angle, measured in a second task. Our model also predicts the results of a categorical reasoning task about changes in the triangle size and shape even when such completion strategies need not be invoked. Taken together, our findings suggest a critical role for noisy physical processes in our reasoning about elementary Euclidean geometry.

## Introduction

Euclidean geometry lies at the foundation of domains such as mathematics, art, and architecture, and its origins have been debated for millennia. Philosophers from Plato^1^ to Descartes^2^ to Kant^3^, have argued that idealized, abstract geometric entities exist innately in all humans. In contrast, scientists like Helmholtz^4^ and Poincaré^5^ have argued that noisy perceptual experience may instead shape geometric reasoning (for a broader socio-historical account of the development of mathematical reasoning, see Lakatos^6^). These two perspectives reflect an inherent tension in the domain of geometry itself: While geometry’s propositions rely on abstract entities like dimensionless points and infinitely long lines, the points and lines of our physical world are dimensional and finite. When faced with a novel geometric problem, how much do we rely on reasoning rooted in physical representations?

Growing research in the cognitive sciences suggests that simulations of the physical world underlie our intuitive reasoning^7^, even in domains like physics, where formal reasoning is abundant and has a long history. Reasoning by simulation has the benefit of predicting future states of the simulated system in situations where spatial or temporal information is missing or when the current state of the system is uncertain^8–15^.

Given the variability in the environments, experiences, and formal education of individuals across human cultures, recent work has also investigated the universality of the processes that might guide geometric reasoning^16–18^. For example, Izard and colleagues^17^ presented a variety of fragmented planar triangles to individuals from a remote Amazonian group, who receive no formal education in geometry and who have no specialized geometric vocabulary. The researchers asked the participants to point to the location of a triangle’s missing corner and to generate its angle using their hands or a goniometer. The Amazonian adults produced responses that were similar to those of formally educated adults in the U.S. and France and that roughly reflected Euclid’s proposition 32, which states that the internal angles of a triangle sum to a constant, regardless of the triangle’s overall size. Nevertheless, 6–7-year old U.S. children given the same task produced responses that appeared to depend on the implied triangle’s overall size, which runs contrary to Euclid’s proposition^19^. In these experiments, only a limited range of triangle sizes was tested (with triangle side length varying by less than 3-fold), and the number of Amazonian participants was necessarily small. Prior work also investigated the effects of extrapolating lines on angle misperception. Weintraub and Virsu^20,21^ show that the intersection of two extrapolated line segments deviates from expectations causing an overestimation of the missing angle (with an exception for small angles which are slightly underestimated). Later, Mitrani and Yakimoff^22^ suggested a theoretical model that studies the effect of variation in base angles. Notably, they discuss the importance of going beyond the mean and to account for higher moments such as the variance of responses. Their model elegantly describes the process of extending straight lines and accounted for the distribution of estimates of the missing vertex^20,21^. However, the size of the base in their experiments varied by only 8 fold, and therefore their model may not be sensitive to changes in the scaling of the distribution with increasing distances. All together, both the experiments with both children and adults from various backgrounds, and the theoretical framework still leaves open the question whether such geometric responses to questions of triangle completion might depend on a dynamic visual routine^23^ or mental simulation^8,9,12^ and how they change developmentally.

In the present study, we address this question using a computational model of a statistical physical process that might guide intuitive geometric reasoning. To do so, we present large samples of educated adults in the U.S. with fragmented triangles and measure the changes in the *distribution* of responses with changes to the size of the triangle. Tasks presented triangle stimuli either as projections on a large screen, which allowed us to test the variation in participants’ responses over large variations in the size of the stimuli, or as images on a computer screen, get large from many participants. Experiments 1–3 investigated the characteristics of participants’ estimates of vertex localization. Importantly, we focus on the statistics of error propagation^24–27^ through an analysis of the probability distribution of vertex estimates over a large range of triangle sizes (varying over 75-fold in size, Experiment 1). We interpreted these results in terms of a dynamic model based on a correlated random walk. To test the model’s validity, we predicted the response of missing angle, and in Experiment 4, tested this against the participants’ estimates of the missing angle. In Experiment 5, we go beyond visual completion tasks to geometric reasoning: We asked a new group of participants to make explicit verbal judgments about the location and angle size of a triangle’s missing corner after verbal descriptions of changes to the other two corners (e.g., “What happens to the angle size of the third corner of a triangle when the other two angles get smaller? Does the third corner angle size get bigger, get smaller, or stay the same?”). Participants could have responded to these questions either using a mental completion process, in which the answers are read off of an imagined, complete triangle or by a general rule about the properties of triangles. Finally, we evaluated whether the model that was fit to the localization data also explained the pattern of categorical responses that we observe in the verbal response task, again aiming to shed light on the role of physical simulation-based mental processes on intuitive geometric reasoning.

## Results

**Figure 1:**
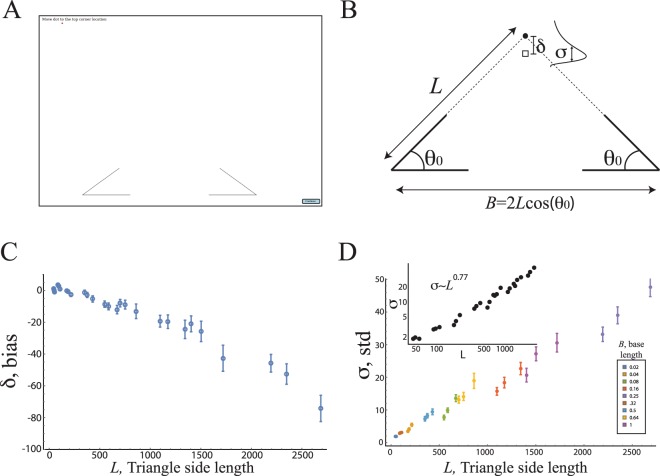
Statistics of the localization of a missing vertex in a triangle completion experiment do not follow Euclidean geometry. (**A**) In the localization task, participants are shown two corners of a fragmented triangle and are asked to position a red dot in the location of the missing third vertex. (**B**) We measured the distribution of participants’ responses to the location of a triangle’s missing vertex over exemplars that varied by ~75 fold in triangle side-length. Side-length values ranged from 44 pixels to 2687 pixels (see Methods) and angle values were 30°, 36°, and 45°. (**C**) The mean deviation (*δ*) from the true y-coordinate location of the missing vertex to the mean of participants’ responses as a function of triangle side-length. Participants’ estimates are biased downwards toward the triangle’s base and scale linearly with triangle side length. (**D**) The standard deviation of participants’ distribution of responses (*σ*) as a function of side length. Inset (log transformed): this standard deviation scales sub-linearly with the side length ($\sigma \sim {L}^{0.77}$, Min-Max values: 0.65–1.04, median exponent: 0.77, 95% CI = [0.73, 0.82]).

In Experiment 1, we asked educated U.S. adults (N = 40) to indicate the location of the missing vertex of 15 different fragmented isosceles triangles (each presented 10 times, all with the base on the x-axis) projected on a large screen (1.07 m × 1.37 m). The side lengths of these triangles varied by 75-fold. With such large variation in triangle size, we were able to analyze the effects of size on the mean and standard deviation of the localization response distribution (Fig. 1A,B).

We found that the y-coordinate localization estimates for the third vertex were biased toward the base of the triangle and that this bias increased linearly with the triangle side-length (Fig. 1C). Strikingly, the standard deviation of the y-coordinate location estimates scaled sub-linearly with side length, $\sigma \sim {L}^{0.77}$ (median exponent: 0.77, 95% CI = [0.73, 0.82], Fig. 1D and Fig. S1). Additionally, while the distribution of the x-coordinate localization estimates also showed a sub-linear scaling of its standard deviation, the errors were 4-fold smaller in magnitude, and while there was a small directional bias at large triangle side length, there was no systematic directional bias up to a 46-fold increase in triangle side length (SI, Fig. S2). We further analyzed response times for the triangle completion task and found that judgments about the localization of the third vertex for smaller triangles were more rapid than for larger triangles (Spearman correlation, *r* = 0.53, *p* < 0.005, see SI, Fig. S3). Thus, processing time in this task is related to the (missing) spatial extent traversed, as is the case for spatial simulation-based processes^9,11^.

To understand these results, we consider various different scenarios. First, if participants are completing triangles using perfectly straight, planar lines (one from each of the two base angles) with no curvature (as on the Euclidean plane), and base angles have a fixed size, then Gaussian noise around the estimated location would show a symmetrical distribution of errors, with the position of the missing vertex averaging to its true location with no bias. Second, if participants are using perfectly straight lines with no curvature, but their assessment of the base angles’ size is fluctuating (as in the Mitrani and Yakimoff model^22^), then we would observe a downward bias towards the base of the triangle. However, since angle variance does not introduce another length scale in the system, this model has no length scale other than triangle side length. Therefore, in a straight line model with variance in the base angles^22^, the standard deviation of the estimates (which has units of length) can scale only linearly with triangle side length. Even if one introduces noise in angle size which is dependent solely on the size of the triangle, the only length scale present would be that of the triangle side length and the scaling of standard deviation will remain linear.

Importantly, any mental simulation process guided by a representation of straight lines, even with added noise from perception and/or action, would result in a standard deviation that scales linearly or super-linearly with the triangle side length (i.e. $\sigma \propto {L}^{n},n\ge 1$, where σ is the standard deviation, *L* is the triangle side length and *n* is the scaling power law). The observed sub-linear scaling of the standard deviation with length precludes the use of Euclidean, straight lines in the localization task (see SI, sections S6, S7 and S15 for more details).

In Experiment 2, we replicated these findings on a large scale with a group of educated adult participants using Amazon Mechanical Turk (N = 100, SI, Fig. S4). Although this replication task differed from the original task in that it only presented triangles differing in side-length size by 25-fold (because stimuli were presented on participants’ own computer screens rather than on our large screen), we observed a similar y-coordinate localization bias toward the base of the triangle and a sub-linear scaling of its standard deviation, $\sigma \sim {L}^{0.65}$ (SI, Fig. S4). Again, participants’ distribution of x-coordinate localizations showed no systematic directional bias, but did show a sub-linear scaling of its standard deviation. Experiment 3 served as a second replication, but presented a rotated version of the task (with the triangle base at the y-axis) to participants on Mechanical Turk (N = 29). This experiment found similar results to the prior two experiments in which the base of the triangle was located on the x-axis (SI, Fig. S5). Thus, our findings cannot be explained by biases in judging the vertical properties of an upright planar shape.

Experiments 1–3 indicate a vertex bias towards the base of the triangle, which is supported by previous research showing a similar error in the judgment of the intersection of two line segments^20,21^. This bias is scale dependent and grows linearly with the size of the triangle. This result could be explained by a mental representation that uses straight lines with noise in the base angles (and thus consistent with a Euclidean representation of space). However, the sub-linear scaling of the standard deviation with triangle side length indicates the existence of another length scale. The additional length scale is at odds with a Euclidean representation of a flat plane.

**Figure 2:**
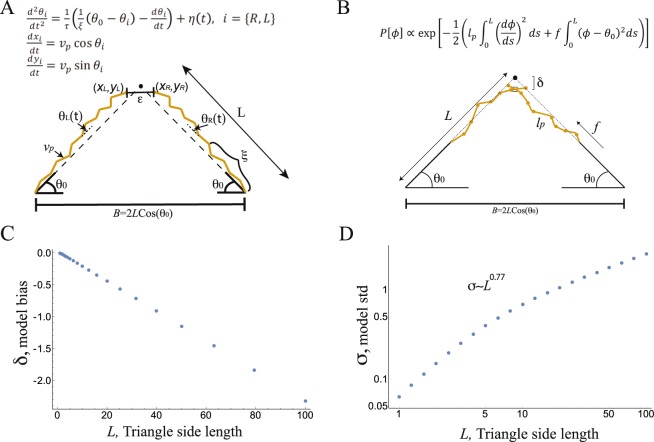
A correlated random walk model captures the results in the triangle completion localization task. (**A**) The schematics of the dynamic model of the triangle completion task based on a correlated random walk. In this model, the local angle evolves with accompanying noise as the line is extrapolated. The model parameters are: *v*_*p*_, a characteristic speed with which the coordinates progress, *ξ*, a time scale for global error-correction (illustrated as number of segments between error-correction events), and *η*(*t*), a noise term with noise amplitude *D* ($\langle \eta (t)\eta (t^{\prime} )\rangle =D\delta (t-t^{\prime} )$), not shown in the figure. The stopping threshold is denoted, ${\epsilon }$, and the base angle is denoted by *θ*_0_. The right and left extrapolation events are simulated independently and are not necessarily symmetrical. The dynamic model converges over many iterations to the statistical model (see SI for further details). (**B**) The schematics of the statistical model. Estimate for the location of the missing vertex arises from balancing noisy estimates of local orientation with a global error-correction mechanism based on the size of the base angles. The model parameters are: *l*_*p*_ the persistence length that penalizes local angle deviations, and f that penalizes global orientation deviations from the base angle *θ*_0_, yielding a correlation length, $\xi =\sqrt{{l}_{p}/f}$, as a fitting parameter. (**C**) The results of a statistical model of triangle completion based on a dynamic random walk show that y-coordinate location estimates have a bias toward the base of the triangle that increases linearly with the triangle side length. Here, we use *ξ* = 2 times the smallest side length, angle noise level *V*_0_ = (*l*_*p*_*f*)^−0.5^ = 0.26 and side length varies between 1–100 (see Methods and SI). (**D**) Using the same parameters as in A), the model also shows that the standard deviation of the y-coordinate location estimate varies sub-linearly with side length, $\sigma \sim {L}^{0.77}$.

To understand how the standard deviation shows sub-linear scaling with triangle side length (and thus a curved representation of space), we created a model for how the fragmented triangle may be completed, inspired by the dynamics of a correlated random walk^28,29^. In this model, participants’ extrapolation of the missing sides of a triangle is described by a set of short concatenated line segments that start at the bottom two vertices, with a given local orientation, and continue until they intersect (Fig. 2A), with repeated corrections to the overall orientation occurring over a time scale *ξ*. The dynamical equations for this process that describe the location of the tip of the line *(x(t), y(t))* that makes an angle *θ*(*t*) with the horizontal are (see Fig. 2A):

1 $$\frac{{d}^{2}\theta }{d{t}^{2}}=\frac{1}{\tau }(\frac{1}{\xi }({\theta }_{0}-\theta )-\frac{d\theta }{dt})+\eta (t)\,$$

2 $$\frac{dx}{dt}={v}_{p}\,\cos \,\theta $$

3 $$\frac{dy}{dt}={v}_{p}\,\sin \,\theta $$

where the parameters of the model are: *τ*, an inertial relaxation time scale for local smoothness, *v*_*p*_, a characteristic speed, *ξ*, a time scale for the global error-correction based on the bottom two angles, and *η*(*t*), a noise term with noise amplitude *D*, ($\langle \eta (t)\eta (t^{\prime} )\rangle =D\delta (t-t^{\prime} )$). In addition, the model has a threshold for the x-coordinate distance between the two extrapolated lines, ${\epsilon }$, which once crossed, ends the process (SI, Figs S6–S8). The right and left extrapolation events are taken to be independent and not symmetrical (symmetry may arise if the triangles are symmetrical rather than from the process itself, see sections S6–S7 in the SI).

While this dynamic approach provides an appealing picture for the mental process of triangle completion, it depends on four different parameters. To reduce the number of parameters, we use the fact that participants were given unlimited time to respond and consider next an equivalent statistical model that ignores time. Repeated implementations of the dynamic model produce a probability distribution for the local angles along the extrapolated line. This statistical approach conveys the benefit of having just one dominant parameter and is described below.

Just as in the dynamical model, the statistical model considers the statistics of a line-like object that is built from small segments that reflect the two competing processes described above – maintaining local motion along a smooth curve and correcting the global extrapolation’s direction given a base angle size. Together, these two processes yield the following probability for the angle of each segment of the extrapolated curve, *ϕ*(*s*), with *s* characterizing location along the curve, relative to the initial base angle *θ*_0_ (see Fig. 2B):

4 $$P[\varphi ]\propto \exp \,[-E]=\exp \,[-\frac{1}{2}({l}_{p}{\int }_{0}^{L}{(\frac{d\varphi }{ds})}^{2}ds+f{\int }_{0}^{L}{(\varphi -{\theta }_{0})}^{2}ds)].$$

Thus, trajectories with higher dimensionless energy (denoted *E)* are exponentially less probable. The form of *E* is exactly the Hamiltonian for a model describing semi-flexible polymers^30–32^, and it has also been used to explain properties of animal navigation^33–35^. We note that in the statistical model, the restriction on a line’s length is directly set by the length parameter, *L*, which sets the upper limit of the range of the integral. This model balances the competition between local and global orientational order. Indeed, the first term reflects the penalty associated with high curvature with a weight known as the persistence length *l*_*p*_, which defines the magnitude of the local noise in the angle judgment at each segment. The second term reflects the penalty for angle deviations from the initial base angle *θ*_0_, with a weight *f*, which acts as a global error-correction mechanism. The model effectively has one parameter, a correlation length $\xi =\sqrt{{l}_{p}/f}$, which balances the two competing effects (and which is proportional to the time scale of the dynamic model (1) up to a factor of the speed *v*_*p*_). The correlation length quantifies the typical length of the trajectory continuing in a certain direction before the error correction resets the angle of the extrapolation to the base angle value.

The exponent observed in the localization experiment, 0.77, suggests that the global error-correction mechanism plays a more dominant role in participants’ triangle completion (see SI, S7.1). Importantly, this signifies *better* robustness to noise propagation with triangle side length than the linear dependence produced by straight lines with noisy angle estimates ($\sigma \propto {L}^{n},n\ge 1$). Taking ξ ∼ 2 times the smallest initial side length and then varying the side length over a 100-fold range allows us to capture the mean and standard deviation of the distribution of the observed participants’ localization responses (Fig. 2C,D and SI, Figs S6–S8 for the dynamic model and Figs S9–S11 for the statistical model). Our model suggests how a simulation-based process which reflects the balance between local straightness (by smooth continuation) and global orientation (by error correction) can produce a sub-linear scaling of the standard deviation with triangle side length, inconsistent with a classical deterministic representation of the Euclidean plane.

As an independent test of the model’s fit to processes of triangle completion, we evaluated whether it predicted the distribution of a new group of participants’ responses for the magnitude of a triangle’s missing angle. With a fixed correlation length of *ξ* = 2, our statistical model, based on localization judgments alone, predicts both that the mean size of the missing angles is overestimated and increases as triangle side-length increases, and also that the variance of the distribution of angles decreases as triangle side-length increases (Similar predictions are achieved with the dynamic model. See SI, Fig. S13 for the dynamic model and Fig. S14 for the statistical model). Indeed, in Experiment 4 (N = 65, SI, Fig. S14), a new group of online participants were asked to use a goniometer to estimate the missing angle size of 10 instances of 15 different triangles (as in Experiment 1). Participants overestimated the size of the missing angle size, and their overestimations increased with increases in the length of the triangle’s sides (at large base angles of 45° and 60°, Fig. S14). The variance of the response distribution decreased as triangle side-length increased (Fig. S14). We analyzed the response times of participants’ angle estimates and found a significant but weak correlation between response times and triangle side-length (Spearman *r* = 0.07, *p* < 0.03, see SI, Fig. S15). These results suggest that participants’ angle estimates are also scale dependent, again at odds with a Euclidean representation and preclude a possible use of a rule (e.g. Euclid’s proposition 32) to answer the missing angle estimates.

In summary, Experiments 1–4 provide evidence that educated adults solve triangle completion problems by engaging in a dynamic mental simulation to construct the complete triangle by extrapolating the sides from the two visible corners to the third, unseen corner. What role might this simulation process play in more explicit reasoning about the general properties of planar triangles? To explore this, in Experiment 5 we presented participants with a triangle completion task probing their intuitions about the general properties of triangles that could be solved without locating any positions or angles in visual space^19^.

In Experiment 5, we conducted a version of the triangle completion task that required participants to produce categorical, verbal judgments about the distance and angle properties of a triangle’s missing corner after changes to the bottom two corners. Such judgments could be made entirely based on formal, Euclidean rules, e.g., those that describe triangle congruency and similarity. Alternatively, such judgments could also be made by mentally simulating the complete triangle and “reading off the answer” from this simulation. We first evaluated the accuracy and response times of participants’ responses to adjudicate between these strategies.

**Figure 3:**
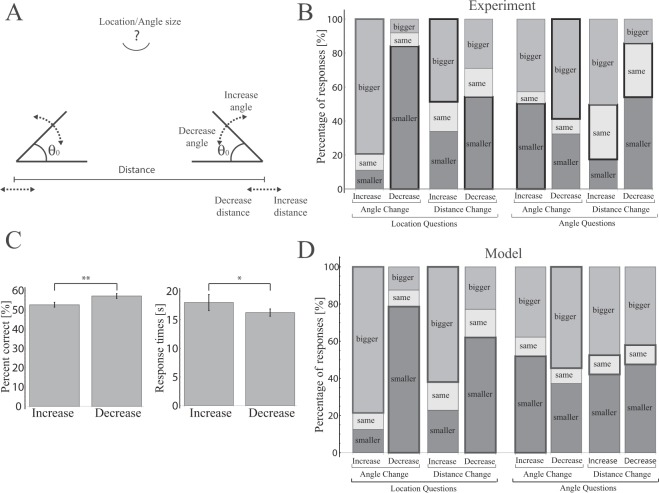
Participants’ responses to a categorical task probing the general properties of triangles. (**A**) In separate blocks of questions, participants were asked to judge the change in the location of the missing vertex and the change in the magnitude of the missing angle. (**B**) Participants’ responses to angle judgments do not adhere to Euclidean rules (correct responses are outlined in bold). For example, participants predominantly judged that an angle should scale with the overall size of the triangle (last pair of bars). (**C**) Participants’ responses are more accurate and faster when triangles get smaller (the angle sizes or the distance between the vertices decreases) vs. larger (the angle sizes or the distance between the vertices increases). Accuracy: Decrease: Mean±STE = 57 ± 1%, Increase: Mean ± STE = 53 ± 1%, Mann-Whitney test: *t*(1628^2^) = 1,264,960, *p* < 0.01, effect size = 0.05; Response times: Decrease: Mean ± STE = 16 ± 1 s, Increase: Mean±STE = 18 ± 1 s, Mann-Whitney test: *t*(1628^2^) = 1,387,910, *p* < 0.02, effect size = 0.05; *p < 0.05, **p < 0.01. (**D**) The statistical model predicts responses similar to participants’ responses in the categorical task shown in (**B**); chi-squared tests with the behavioral data showed values > 0.17. A Bayes factor estimate comparing the model with a model of only noise in base angle estimates with similar thresholds showed values of BF > 10^10^ for most questions (with the exception of AID (Angle question with Increasing Distance) BF = 0.08 and ADD (Angle question with Decreasing Distance) BF = 3). Model parameters are *ξ* = 2, *V*_0_ = 0.4, Th_L_ = 0.05, Th_A_ = 0.05, all initial base angles = 36° and initial side length distance varied between 2–4 (see Methods and SI).

For this experiment, a new group of adult participants on Amazon Mechanical Turk (N = 407) were asked in two separate blocks: whether a triangle’s vertex would move up, move down, or stay in the same place after the other two vertices either moved farther apart, closer together, increased in angle size, or decreased in angle size. Participants were also asked whether the associated angle at that third vertex would get bigger, get smaller, or stay the same size after those same four transformations (totaling 8 multiple choice questions with chance at 33%; Fig. 3A). While the participants saw only a static fragmented triangle on the screen with no accompanying visual transformations, they were introduced to the task with visual displays that exemplified each change. We measured accuracy and response times of the participants.

Participants performed well above chance in their location judgments when either angle or distance to the other two corners is changed (Fig. 3B), and in their angle judgments when the other two angles are changed. Nevertheless, their performance was far from perfect, and their angle judgments in response to changes of distance between the other two corners were no better than chance level, with a bias towards the direction of the manipulation (“bigger” for increased distance and “smaller” for decreased distance, Fig. 3B). Erroneous responses to this question were in direct contradiction with Euclid’s proposition 32, which states that the internal angles of a triangle sum to a constant (e.g., participants responded that the missing angle got bigger when the other two corners maintained their angle measure but moved farther apart; Fig. 3B and Fig. S14). Notably, these error patterns accord with the errors made by children in the previous studies of triangle completion^19^ and are qualitatively similar to the performance of U.S. and Amazonian adults^17^.

While participants’ incorrect responses, e.g., that a triangle’s third angle should scale with the triangle, indicate that they did not follow the rule that the internal angles of a triangle should always sum to a constant, they may have nevertheless used a faulty rule to answer these questions. Evidence against the possibility that participants relied on rules at all during this task comes from a comparison of responses to the questions referring to transformations that decreased vs. increased the side lengths of the triangle. If participants were using a rule (whether accurate or faulty) which is scale invariant, then no such differences should be found. However, if participants were making their responses based on simulated triangles, then there might be greater success and shorter response times after transformations that decreased the triangle side lengths since the simulation process would be shorter and entail less noise accumulation. Our results show that participants responded more accurately and in less time after they were asked to make judgments about triangles whose side lengths got shorter vs. longer (Accuracy: Mann-Whitney test: *t*(1628^2^) = 1,264,960, *p* < 0.01, effect size = 0.05; Response Time: Mann-Whitney test: *t*(1628^2^) = 1,387,910, *p* < 0.02, effect size = 0.05; Fig. 3C), consistent with a strategy based on imagery which is not scale invariant.

Our statistical model described above and characterized in Experiments 1–4 relies only on the dynamic properties of participants’ localization of the missing third vertex of a triangle, given fragmented information about the other two corners. Here we ask whether the model can nevertheless capture the results obtained in the categorical triangle completion task of Experiment 5. For example, for a question asking what happens to the missing vertex location after an increase to the distance between the two base vertices, we approximated the distribution of the vertex location in the initial distance case by a Gamma distribution with the corresponding model values of the mean and variance in estimates and then compared it to a Gamma distribution with parameters taken from the increased distance case. Thresholds for the “move up”, “move down”, and “stays the same” categories were set according to the ratio of the measured bias and standard deviation of participant location estimates from Experiment 1 (where participants localized the third vertex over 75-fold changes in triangle side-length; see Methods and SI).

We found that our model produced responses that closely resembled those of the participants in the categorical task for all 8 questions (Chi squared tests, all *ps* > 0.17, Fig. 3D vs. Figure 3B). Furthermore, comparing the model’s predictions to another model’s prediction, which included only noisy estimates of the base angle sizes, yielded better support for our model (Bayes factors^36^ (BF) for most questions >10^10^, and AID BF = 0.08 and ADD BF = 3, see Methods and SI). The largest deviation between the model’s predictions and participants’ responses was in those questions where the angle size of the third location was probed after changes to the distances between the bottom two corners: The model under-predicts the number of accurate responses. The current results do not reveal whether the greater number of participants who succeeded in this question did so because of less noisy simulation strategies for this pair of questions or because of invoking a rule of some kind. While there were too few individuals who responded correctly to these questions to investigate their consistency and reaction times compared to the other participants in the study, future work might investigate how differences in accuracy and reaction time relate to different responses strategies across individuals. Years of formal schooling, however did not significantly predict accuracy on the categorical task (Spearman correlation median *r* = 0.002, 95% CI = [0,0.28], see Fig. S16), consistent with the studies comparing educated to uneducated participants in the U.S. and the Amazon^17^. We note that when we vary model’s parameters for each question or add another parameter to denote noise in the base angle estimates, the model fits the behavioral results even better (see SI, Figs S17–S20).

## Discussion

While previous studies have shown that intuitive geometric reasoning is universal in adults across cultures and levels of education^17^, the mental processes and representations that might guide this reasoning remain unclear. Our study provides both behavioral evidence and a computational framework showing how intuitive geometric reasoning about planar triangles in adults depends on the mental simulation of locally correlated motion along a line segment and the correction of that accumulated motion’s global direction. While our work does not preclude the existence of Euclidean geometry considerations in geometric reasoning, it quantifies it in terms of physically measurable parameters. For example, the global orientation demand is the dynamic equivalent of the Euclidean demand for a globally straight line, while the local smoothness condition is associated with the local definition of a straight line. Thus the mental simulation process that balances local smoothness and global orientation leads to an intrinsic length scale that controls the nature of geometric reasoning that is a noisy variant of classical Euclidean geometry.

Might there be an advantage of such a dynamic strategy for geometric reasoning? While a static Euclidean representation like “lines are straight,” or “the internal angles of a polygon sum to a constant” may provide the most accurate and rapid response to a question about a planar shape, its perceptual implementation to stimuli might be hard to determine, if, for example, the question refers to a shape with noisy, indeterminate properties (e.g., a situation in which it is difficult to tell whether the judgment should be about a line vs. a curve embedded on a plane or on a sphere). Simulation-based strategies, however, can be more robust to noise, with smaller variations in estimates around similar or indeterminate conditions. In the present study, the standard deviation of participants’ localization errors scaled sub-linearly with triangle side-length. This sub-linear dependence was smaller than the linear (or higher) dependency that would be expected by the noise accumulating around straight lines. As such, relying on one straight line from each base angle actually leads to worse performance than the one obtained on the present localization task. Thus, participants’ adoption of a method that balanced local smoothness with a global angle correction served as a better strategy to preserve essential shape properties given the physical constraints of the problem. Indeed, such a process still produced accurate estimates of the location and angle size of fragmented triangles’ missing third corners. The use of mental simulations that lead to robust, Euclidean-like estimates to geometric questions in noisy situations may reflect the mature geometric intuitions that universally guide our reasoning.

When asked categorical questions about changes to angle size upon increasing or decreasing of the triangle’s base length participants performed no better than chance. One may thus ask whether a literal representation of Euclid’s proposition 32 is needed to answer this type of question. Drawing from other cognitive processes - we note that individuals without training in linguistics do not have a literal representation of phrase structure grammar, but they use it intuitively and automatically in speaking and in understanding the speech of others. Similarly, children who judge that one can count on from any number, however high it is, surely do not have a literal representation of Peano’s axioms, yet their judgments accord with them^37^. Thus, we need not assume that people would need to have an explicit knowledge of proposition 32 to judge automatically that if the scale of the triangle changes, then its shape (and therefore its three angles) remains unchanged. In our experiments, we recorded years of education as an indirect measure of mathematical proficiency, which showed no correlation with participants’ accuracy. Yet this leaves open the question of whether participants’ mathematical proficiency in different fields such as mathematics, the visual arts, and architecture, could account for their judgments.

Further work is also needed to understand the specific perceptual and neural mechanisms underlying the mental simulation process. The simulation process employs line extrapolation with two competing constraints: local smoothness and global orientation. On the perceptual level, studies suggest that local smoothness can be extracted by curvature measurements by receptive fields^38,39^. This may reflect the ability of our visual system to follow a smooth trajectory locally, similar to the well-known gestalt principle of ‘good continuation’^40–46^. Angle error-correction may reflect a high-level capacity of short term visual memory to represent global orientation^28,29,47–50^. Connecting developmental work, eye tracking, and brain activity measurements with people’s estimates in the triangle completion task, would serve to elucidate the determinants of the correlation length ξ that suffices to capture the statistics of our vertex localization task, missing angle estimates, and even categorical reasoning.

Our work contributes to accumulating evidence that statistical dynamic strategies may underlie foundational reasoning capacities that may otherwise appear rule-based and static, especially in domains like physics, in which intuitive reasoning relies on models of the world that are unfolding in time^7–15^. Our model extends previous simulation-based models^7–15^ in two essential ways. First, contrary to simulation models that introduce noise as variation in the estimated physical parameters, our model focuses on the propagation of errors and balances two competing error-control demands: local smoothness and a global error correction. These two competing demands govern the process which guides our judgments of geometry. Second, most simulations consider transformations in space and time on existing representations. In contrast, our simulation model constructs the mental representation itself, i.e. the geometric shape. Further work is needed to disentangle the relative role of these processes. This becomes particularly apparent in the context of illusions, e.g. Kanizsa triangle, which require the reconstruction of geometric shapes. Examining the robustness of the constructed mental representation to changes of scale, and inferring the role of errors/deviations in triangle corners’ position and corners’ angle-size, can illuminate better the characteristics of geometric completion simulations^51–53^.

Our model serves as a first approximation to the salient features that might guide geometric completion processes. For example, it assumes a robust evaluation and memory of angle sizes, which allow the global error correction process to dominate. It is possible, however, that throughout the process of line extrapolation, this angle size representation is degraded and the error correction mechanism gets noisier. Future models can test for such degradation effects, addition of noisy distributions of initial estimates of base angles (see SI for a treatment of Gaussian noise in base angles), angle estimates dependence on their orientation (as found in refs^20,21^), and noisy estimates of other model parameters (such as the linear length scale (L), the threshold distance between the two extrapolated lines (ε), and the time intervals for error-correction events (ξ)).

Participants’ answers to and response times for the categorical task associated with shape changes suggest that they simulated the properties of complete triangles. Though these questions could have been answered easily and quickly with Euclidean rules, participants did not invoke these rules. Considering the conditions under which participants might invoke geometric or other rules in the presence of additional cues (such as changing the triangle’s orientation, color, or labeling angle sizes), could point to the relative importance of the statistical-dynamics of geometric reasoning. More broadly, our study suggests that geometric pedagogy may benefit from relying more on simulation-based reasoning and finding conditions under which using geometric rules becomes intuitive to improve the learning and application of those rules.

While geometry is often seen as underlying our conception of the physical world, it may also be the case that our perception of the physical world underlies our intuitive geometry. An interesting question about the nature of the mental simulations we use then arises: Do we aggregate our mental simulations to produce an averaged statistical representation of geometry used for a variety of question types, or do we employ a dynamic model every time we are challenged by a question in geometry? Since psychological mechanisms shared by animals, children, and adults allow for perception and navigation in uncertain and imperfectly known environments, how humans have succeeded, over time, to convert reproducible strategies for these tasks into mathematical abstractions and rules is a natural next question.

## Materials and Methods

All experiments in this study adhere to the regulations and guidelines on the use of human subjects. All experimental protocols were approved by the Harvard IRB committee (Committee on the Use of Human Subjects). All participants gave their informed consent to participate in the experiments detailed below.

### Experiment 1 - Localization of the missing vertex in a triangle completion

In a laboratory experiment, we showed participants 15 different incomplete isosceles triangles 10 times in a random order (for a total of 150 triangles for each participant). Forty participants, divided randomly into two equal-sized groups, were shown triangles of 3 different base angle sizes (30, 36, and 45 degrees) and with 5 different base lengths. Participants in group 1 were shown base lengths of 0.02, 0.08, 0.25, 0.5, and 1. Participants in group 2 were shown base lengths of 0.04, 0.16, 0.32, 0.64, and 1. In both groups, 1 signifies 1900 pixels and is equivalent to 130 cm. Participants sat at a distance of 150 cm from the screen. For each triangle, we asked participants to position a dot in the estimated location of the missing vertex. Before the experiment began, participants had one practice trial, in which the location of the missing vertex was indicated by a dot of a different color, and they were asked to position their dot on the indicated position. We consider the triangle side length as the primary variable in the analysis since our model of line extrapolation points to this quantity as the length scale of the computation process. Regression analysis of the bias and standard deviation dependence on base angles and base length further indicated a robust and significant effect only for the interaction term of base length and base angles (i.e. the side-length, Bias: base length *p* > 0.4, base angle *p* > 0.75, base length * base angle *p* < 0.0001, Standard deviation: base length *p* > 0.47, base angle *p* > 0.53, base length * base angle *p* < 0.00001).

### Experiment 2 - Localization of the missing vertex in a triangle completion task

In an online experiment (Amazon Mechanical Turk), we repeated the same task as in the lab experiment with 100 participants, divided into two groups. Base angles were set to 3 different angle sizes 30, 45 and 60 degrees – group 1 (50 participants), and 36, 51, and 66 degrees – group 2 (50 participants), with 5 different base lengths of 0.1, 0.25, 0.5, 0.75 and 1. Since triangles would have exceeded the size of the screen with the angle sizes presented in group 2 at the distance scale used in group 1, group 1 saw a y-coordinate length scale of 900 pixels and group 2 saw a y-coordinate length scale of 1300 pixels. To match the scales for the two groups we divided the estimates of the second group by a ratio of 13/9.

### Experiment 3 - Localization of the missing vertex in a triangle completion task

We repeated the same online task of positioning the missing vertex with a rotated isosceles triangle such that the base of the triangle was on the vertical axis, on the right side of the screen. Twenty nine participants were shown 3 different base angle sizes (30, 45, and 60 degrees), with 5 different base lengths (0.1, 0.25, 0.5, 0.75, and 1), where a base length of 1 was set to be 1000 pixels.

### Experiment 4 - Estimation of missing angle in a triangle completion task

In an online experiment, we asked participants (N = 65, Amazon mechanical Turk) to estimate the missing angle size in a triangle completion task. Participants moved a slider to set the angle size of a fragmented triangle. The slider and angle were located at the top right side of the screen, away from the fragmented triangle. Base angles were set to 3 different angle sizes 30, 45 and 60 degrees, with 5 different base lengths of 0.1, 0.25, 0.5, 0.75, and 1, where a base length of 1 was set to be 1000 pixels.

### Experiment 5 - Categorical geometric reasoning experiment

In an online experiment (Amazon mechanical Turk) we asked participants to answer 8 randomly ordered categorical questions regarding imagined manipulations to triangle size or shape. Participants were presented with the two base corners of an incomplete isosceles triangle and were asked what would happen to the location (or angle size) of the missing vertex upon an increase (or decrease) of 20% in the distance between (or angle size of) the two bottom corners. Participants saw the same drawing of a static, fragmented triangle with each question throughout the experiment. In different groups of participants, this accompanying triangle had corners that were either 600 pixels and 240 pixels apart and presented either 36 and 60 degree angles. Each experiment started with a demonstration of what the indicated manipulations to distance and angles of the base corners looked like on a different example triangle. For each imagined manipulation, participants indicated whether the missing corner’s location would move up, move down, or stay in the same place. Similarly, they also indicated whether its angle size would get bigger, get smaller, or stay the same size. Four-hundred-seven participants completed the experiment: 157 females; 247 males; and 3 who did not specify a gender. Participants’ age ranged between 18–72 years, with a median of 31 years. Participants’ years of education ranged between 8–33 years, with a median of 15 years (and see SI, Fig S16).

### Analysis of all behavioral data

All data analyses were done using Mathematica 11.0. The mean deviation from the true location of the missing vertex or the missing angle size, and the standard deviation were calculated for each participant and then averaged across participants. Results in the main text show mean ± std.

### Derivation of Y-coordinate mean and variance

In order to model and predict the quantitative results for the localization task, we assumed the estimated location (*X*, *Y*) was the average intersection of a right and left triangle’s side trajectory extrapolations (see Fig. S7). Using the statistical model, we derived analytic expressions for the moments of each side extrapolated trajectory by using $x={\int }_{0}^{L}\,\cos (\varphi (s))ds$ and $y={\int }_{0}^{L}\,\sin (\varphi (s))ds$ where *ϕ*(*s*) were taken from the probability distribution: $P[\varphi ]\propto \exp \,[-\frac{1}{2}({l}_{p}{\int }_{0}^{L}{(\frac{d\varphi (s)}{ds})}^{2}ds+f{\int }_{0}^{L}{(\varphi (s)-{\theta }_{0})}^{2}ds)]$. We calculated the bias in the location estimate by subtracting the true location (*y*_*true*_ = *LSin*[*θ*_0_]) from the mean y-coordinate. The standard deviation was calculated as the square root of the second moment of the distribution of the estimated (*X,Y*) location. The correlation length, $\xi =\sqrt{{l}_{p}/f}$, is the dominant parameter setting the scaling exponent between the vertical location standard deviation and the side length. We found a best-fit to the participants’ responses at a value of *ξ* = 2 (where 1 denotes the side-length of the smallest triangle considered), and the side length varies across *L* ∈ [1,100] (a similar range as the experimental setup). A detailed calculation of the moments and sensitivity analysis of model parameters are presented in the SI. And see Fig. S21 for the relation between base angle and error in the estimated mean location of the missing vertex. We also derived the distribution of the mean and variance using the dynamic model by simulating Eqn. (1–3), which resulted in similar distributions (see SI for more details).

### Model estimate of the statistics of the missing angle

The magnitude of the missing angle was calculated using estimates of the missing vertex. Given the good fit of the Gamma distribution to participants’ y-coordinate estimates in the localization task (see SI, Fig. S12), we approximated in our model the vertical coordinate distribution as a Gamma distribution whose mean and variance were derived from our model’s analytical calculations. The x-coordinate was sampled from a Gaussian distribution with a standard deviation derived from the same analytical calculations. This produced a set of (*X,Y*) locations that was used to derive the estimated missing angle size value. The missing angle size was calculated as: Missing angle size = π − (effective base angle right + effective base angle left). We repeated this process 400 times to produce a distribution of estimated angles per side length and base angle. We then calculated the mean and standard deviation as a function of side length and base angle. We used the same correlation length, *ξ* = 2, and noise levels of *V*_0_ = 0.4. The dynamic model simulations of Eqns (1–3) yielded similar results, see SI for a detailed description.

### Model estimates of the categorical geometric reasoning task results

The categorical geometric reasoning task of triangle completion challenged participants to compare location or angle size estimates from two triangles, an initial incomplete triangle presented on the screen and an imagined triangle resulting from a specific manipulation (increasing or decreasing the distance between or angle size of the two base angles; see above). We thus compared the model’s predictions for the locations or angles of the initial triangle to the triangle that would result from the indicated manipulation. For example, consider a question about the location change of the missing vertex after an increase of the distance between the two base vertices. We calculated the location estimates of the model for the initial triangle by plugging the model’s predicted mean and variance to a Gamma distribution yielding a sample of 400 estimated locations (see SI, Fig. S12). We then repeated this process for the manipulated triangle. Next, we compared the two samples of location estimates in pairs. For each pair, we calculate the percent change in location, and used a threshold to categorize the answer as “move up”, “move down” or “stays in the same place”: Locations which were 5% higher than the initial estimated location we marked as “move up” $(\frac{{y}_{{\rm{after}}}-{y}_{{\rm{init}}}}{{y}_{{\rm{init}}}} > 0.05)$. Locations which were 5% lower than the initial estimated location we marked as “move down” $(\frac{{y}_{{\rm{after}}}-{y}_{{\rm{ini}}t}}{{y}_{{\rm{init}}}} < -0.05)$. All values in between these two thresholds were marked as “stays in the same place” $(-0.05 < \frac{{y}_{{\rm{after}}}-{y}_{{\rm{init}}}}{{y}_{{\rm{init}}}} < 0.05)$. This categorization method was also used with angle questions. The threshold was set by estimating the median coefficient of variation (std/mean) in participants’ answers in the localization task of the missing vertex location estimates (See SI for more details and sensitivity analysis of the thresholding values). For all questions the following parameters were used: correlation length, *ξ* = 1.25, variance of interior angles estimates *V*_0_ = 0.5, initial side-length, L = 3.2 for location questions and L = 1.25 for angle questions, initial angle = 36 degrees, increased angle = 45 degrees, length increase for location questions = 25%, length increase for angle questions = 50%. Similar to previous sections, we also compared the dynamic model simulations with the categorical behavioral responses, yielding similar results to the statistical model (see SI for more details).

### Goodness of fit for the model and the categorical geometric reasoning task results

We used a Chi-Squared test for goodness of fit between the model predictions and the participants’ responses in the categorical geometric reasoning task. These tests did not show a significant difference between the model and participants’ responses. Chi-Squared statistics and p-values were: t(1) = (1.87, 1.87, 1.33, 1.33, 1.33, 0.75, 0.14, 0.14), *p* = (0.17, 0.17, 0.25, 0.25, 0.25, 0.39, 0.7, 0.7) for VIA,VDA, VID, VDD, AIA, ADA, AID, ADD questions respectively (each condition first letter indicates whether the question concerned vertex location or angle size (V/A), the second letter indicates whether the manipulation concerned increase or decrease in value (I/D) and the third letter indicates whether the manipulation suggested concerned changes to the base angles size or the distance between the base angles (A/D)).

### Bayes factor comparison of the model and a model with only noisy base angle estimates

We used Bayes factor analysis to validate the fit of our model to the categorical geometric reasoning task results. We compared our model with a model that used straight lines with only Gaussian noise in the assessment of the base angle - angles were assumed to be sampled from a Gaussian distribution with the mean set to the base angle and a standard deviation of 5 degrees (see SI for more details). We used the same thresholds for both models (5% change as a detection threshold). The Bayes factor was calculated as

$$BF=\frac{P(D|\mathrm{WLC}\,\mathrm{model})}{P(D|\mathrm{trig}\,\mathrm{model})}={(\frac{{P}_{{\rm{WLC}},{\rm{smaller}}}}{{P}_{{\rm{trig}},{\rm{smaller}}}})}^{{n}_{{\rm{smaller}}}}{(\frac{{P}_{{\rm{WLC}},{\rm{same}}}}{{P}_{{\rm{trig}},{\rm{same}}}})}^{{n}_{{\rm{same}}}}{(\frac{{P}_{{\rm{WLC}},{\rm{bigger}}}}{{P}_{{\rm{trig}},{\rm{bigger}}}})}^{{n}_{{\rm{bigger}}}},$$

where P_WLC,i_ are the probabilities derived from our model, P_trig,i_ are the probabilities derived from a model with only noisy base angle estimates and n_i_ are the number of such responses in the categorical geometric reasoning task. The BF results were BF = (10^27^, 10^18^, 10^119^, 10^96^, 10^62^, 10^147^, 0.08, 3) for the VIA,VDA, VID, VDD, AIA, ADA, AID, ADD questions respectively, indicating that, for most questions, the WLC model is superior to the simpler, straight-line Euclidean model with noisy base angle estimates.

### Data availability

The experimental data is available online on the following link: https://github.com/StatShapeGeometricReasoning/StatisticalShapeGeometricReasoning with the data files. Please refer to the README file for explanations on the data structure of each file:

1. Experiment 1: exp1data.csv
2. Experiment 2: exp2data.csv
3. Experiment 3: exp3data.csv
4. Experiment 4: exp4data.csv
5. Experiment 5: exp5data.csv.

## Electronic supplementary material

Supplementary Information
