# Supplementary material for: The statistical shape of geometric reasoning
Source: Sci Rep. 2018 Aug 27;8:12906. doi: 10.1038/s41598-018-30314-y (PMC6110727; doi:10.1038/s41598-018-30314-y)
Supplement: Supplementary file 1 — Supplementary Information [file 41598_2018_30314_MOESM1_ESM.pdf]

The statistical shape of geometric reasoning – Supplementary Information

Y. Hart, M. R. Dillon, A. Marantan, A. Cardenas, E. Spelke, L. Mahadevan

Table of Contents

Additional Data and Analysis **2**

S1 The distribution of the individual scaling exponents showed a sub-linear range **2**

S2 X-coordinate shows a small right bias and standard deviation that scales sub-linearly with side length **2**

S3 Response times of participants in the localization task correlate with triangle's size **2**

S4 Online localization experiment shows similar results to the laboratory experiment **2**

S5 Rotating the triangle does not show a preferential bias that differentiates the vertical from the horizontal completion tasks **3**

S6 Dynamical model equations and sensitivity analysis **3**

S7 Statistics deduced from the local-global mathematical model **4**

S7.1 Bounds on the scaling exponent for locally dominated and globally dominated strategies 5

S7.2 The three moments of the y-coordinate distribution for the complete model 6

S7.3 Calculation of the predicted moments with a noisy base angle 9

S8 Sensitivity analysis of statistical model's moments to parameter variations **10**

S9 Fitting experimental estimates in the localization task of the missing vertex to a gamma function distribution **11**

S10 The model predicts the observed variations in the estimates of the missing angle: overestimate increases and variability decreases as side length increases **11**

S11 Response times of participants in the missing angle task correlate with triangle's size **12**

S12 Years of education have no effect on the accuracy of responses in the categorical geometric reasoning task **12**

S13 A "trigonometric" null-model for the categorical geometric reasoning task **13**

S14 Sensitivity analysis of the model predictions in the categorical geometric reasoning task **15**

S15 The statistics of the location of the missing vertex as a function of the base angle **16**

Figures **18**

Additional Data and Analysis

S1 The distribution of the individual scaling exponents showed a sub-linear range

Each participant was shown 15 different triangles, composed of one of 5 base lengths and one of 3 angle sizes; each triangle was presented 10 times in a random order. We calculated the scaling exponent of the Y-coordinate standard deviation with the side length, $\sigma = L^n$. We found that the scaling exponents, n, ranged between 0.65-1.04 and peaked at 0.77, indicating a wide range of scaling exponents, mostly sub-linear. Figure S1 shows the histogram of the different scaling exponents.

S2 X-coordinate shows a small right bias and standard deviation that scales sub-linearly with side length

We note that contrary to the vertical location of the missing vertex, the horizontal (x-axis) estimate was likely influenced by the symmetry of the two identical vertices shown on the screen. We found that the x-coordinate estimates of the missing vertex were close to the true location with an increase of the bias toward the right for only large values of the side length (Fig. S2A). The maximum bias magnitude was 4-fold smaller than the maximum bias found for the y-coordinate.

We also calculated the dependence of the standard deviation of the x-coordinate with triangle side-length and found that it scales sub-linearly with side length, with a scaling exponent, n, of 0.86 (median exponent=0.86, 95% CI=[0.84,0.87], Fig. S2B). The variance in the x- coordinate was ~2-fold smaller than the one observed for the y-coordinate.

S3 Response times of participants in the localization task correlate with triangle's size

In order to assess the similarity of the triangle completion localization task to other simulation-based phenomena, such as mental rotations, we analyzed the response times of participants as a function of triangle size. We find that the mean response time significantly correlates with triangle's side length (Spearman correlation r=0.53, p-value<0.005, Fig S3A). A second analysis with all response times (not averaged across all participants) still shows a correlation with triangle's side length (Spearman correlation r=0.22, p-value<10^{-7}, Fig S3B).
The correlation of response times with triangle's side length is thus reminiscent of other simulation-based processes, such as mental 3d rotation.

S4 Online localization experiment shows similar results to the laboratory experiment

To validate our results, we repeated the laboratory experiment with an online experiment on Amazon's mechanical Turk platform. One-hundred participants completed the online study and

were randomly assigned to one of two groups presenting different experimental parameters. All participants were shown 15 triangles, at base lengths of 0.1, 0.25, 0.5, 0.75, 1 of the maximum base length (which was 900 pixels for group1 and 1300 pixels for group 2). Each base length presented 3 different angle sizes (30, 45 and 60 degrees for group 1 and 36, 51 and 66 degrees for group 2). Each triangle was presented 10 times, totaling 150 trials. The total change in triangle side-length ranged 21 folds.

We found similar results to the laboratory experiment: The error in the mean estimate was biased toward the base, increasing linearly with side length (Fig. S4A). The standard deviation scaled sub-linearly with the side-length, with a mean scaling exponent of 0.64: $\sigma \sim L^{0.64}$ (Fig S4B). While it is not clear why the scaling exponent for our lab experiment, which dealt with much larger triangles, was larger than that of the online study, the increase in the exponent is consistent with the difficulty of tracking the initial base angle over long distances and a possible increase in the variance of interior-angles estimates (V_0^2) in the online experiment (see section S8 for more details).

S5 Rotating the triangle does not show a preferential bias that differentiates the vertical from the horizontal completion tasks

We checked whether our observations were influenced by an inherent bias present along a vertical axis. To do this, we rotated the original triangle by 90° so that the missing vertex lies along the x-axis, while the base lies along the y-axis (Fig S5A), and then asked participants to locate the missing vertex. The responses reflected a mean location that was proportionally displaced towards the base of the triangle, except when the missing angle was 30 degrees (Fig. S5B). Furthermore, the standard deviation scaled sub-linearly with the side length, with $\sigma \sim L^{0.66}$ (Fig. S5C), but its magnitude was 1.6-fold larger (compare with Fig. S4).

S6 Dynamical model equations and sensitivity analysis

To model the mental process for the triangle completion task, we start with a dynamical model. The model describes the evolution in space and time of two trajectories, launched from the vertices along the triangle base (see Fig S6). The equations for the evolution of each trajectory (*i*=R,L)

(S1) $$\frac{d^2\theta_i}{dt^2} = \frac{1}{\tau}\left(\frac{1}{\xi}(\theta_0 - \theta_i) - \frac{d\theta_i}{dt}\right) + \eta(t), \;\; i = \{R, L\}$$

(S2) $$\frac{dx_i}{dt} = v_p \cos\theta_i$$

(S3) $$\frac{dy_i}{dt} = v_p \sin\theta_i$$

where the model parameters are: τ, an inertial relaxation time scale, v_p, a characteristic speed, ξ, a time scale for global error-correction (which is proportional to the correlation length defined in the statistical model in the main text, up to a factor of the speed v_p), and $\eta(t)$ is a noise term with noise amplitude D ($\langle \eta(t)\eta(t') \rangle = D\ \delta(t-t')$). In addition, the model has the base length, B, as a parameter and a threshold for the x-coordinate distance between the two curves, ϵ, which once crosses, ends the process. The current model does not consider possible variations in the base-angle estimates, θ_0, which could be another parameter in the model.

To explore the individual contribution of each parameter, we varied each parameter of the model while keeping the rest of the parameters fixed (v_p, ϵ, D, ξ, B). Since timing does not play a role in our current experimental setup, we set the integration time scale, $\tau = 1$, throughout all the calculations. Similarly, the base angles were fixed to 45 degrees unless otherwise stated. We present below the sensitivity analysis of the dynamic model statistics (mean and variance) to the model parameters as well as the predicted angle estimates from the dynamical model.

As expected, the scaling exponent, n, highly depends on the error-correction time scale, ξ, ranging from 0.5 for small values of ξ and increasing above 1 for high values of ξ, indicating a transition between the error-correction mechanism to the correlated random walk regime (Fig. S7A). Similarly, the speed, v_p, has the same effect on the scaling exponent: as it increases, the scaling exponent increases (Fig. S7B). The interior-angle noise level, D, decreases the observed scaling exponent mildly over a range of almost two orders variation (0.01 to 1, Fig. S7C). Lastly, increasing the stopping criteria threshold, ϵ, increases the scaling exponent (Fig. S7D).

The mean deviation from the missing vertex true location (δ) shows a strong dependence on the normalized stopping criterion (Fig. S8A). The interior angles noise level (D), error-correction time scale (ξ), and speed (v_p), play a secondary role in the mean deviation results (Fig. S8B-D, compare with the results of the statistical model).

S7 Statistics deduced from the local-global mathematical model

In the following three subsections, we present a derivation of the statistical properties derived from the mathematical model associated with the balance between local smoothness and global reorientation. We start in section S7.1 with a derivation of the upper and lower bounds on the scaling exponent, n, that links the standard deviation of the location of the missing vertex and the side-length. In section S7.2 we derive expressions for the first three statistical moments of the distribution of the vertical location of the missing vertex as a function of the correlation length in our model. In section S7.3 we derive expressions for the moments by accounting for noise in estimates of the base-angles as well.

S7.1 Bounds on the scaling exponent for locally dominated and globally dominated strategies

We first consider the case when there is no global error correction that causes the trajectory to hew to the base angle. Instead, we assume that extrapolation is achieved by keeping two consecutive angles close to each other (with a corresponding penalty on bending of the curve). This mechanism tends to maximize smoothness, and in this case, the dominant term in statistical model (Eq. (2) in the main text) is

(S4) $$l_p \int_0^L \left(\frac{d\phi}{ds}\right)^2 ds.$$

Writing the angle as, $\phi \sim \frac{\delta}{L}$, where L is the length of the extrapolated line and δ is the standard variation of the y position of the line, if the overall amplitude of the fluctuations can be assumed to be in statistical equilibrium, this implies that

(S5) $$l_p \left(\frac{\delta}{L}\frac{1}{L}\right)^2 L \sim 1$$

and so

(S6) $$\sigma = \langle \delta^2 \rangle^{1/2} \sim L^{3/2} l_p^{-1/2}$$

i.e. the standard deviation of the location scales with side-length to the power $n = 3/2$. To understand this, we note that for a rigid line with fluctuations only in the base angle (see also sections S7.3 and S15), one expects linear scaling of the standard deviation with the length. When using short noisy segments, there is an additional accumulation of noise at each "step" in the extrapolation process, so that the exponent should be larger than 1.

In the other limit, two consecutive angles are no longer locally correlated but rather hew to the global base angle. Intuitively, this is a limit of a random walk and one expects the scaling exponent to be 1/2. Following the same considerations as those for the smoothness term, one has

(S7) $$f \int_0^L (\phi - \theta_0)^2 ds$$

(S8) $$f \left(\frac{\delta}{L}\right)^2 L \sim 1$$

(S9) $$\langle \delta^2 \rangle^{1/2} \sim L^{1/2} f^{-1/2}$$

which indeed result in a scaling exponent of $n = 1/2$.

The two limiting mechanisms serve to bound the scaling exponent, n, to lie between 1/2 and 3/2. The vertical location estimates in the localization task show that the scaling exponents range between 0.65-1.04 with a median of 0.77 (Fig. S1), suggesting a slight dominance of the global error-correction mechanism over the local smoothness of the extrapolation.

S7.2 The three moments of the y-coordinate distribution for the complete model

The statistical model assumes a probability distribution that penalizes both large changes in local angle estimates (smoothness) and large deviations from the base-angle value (error-correction). With no loss of generality, we assume the base angle is set to zero by rotating the frame, and assume a constant length, L, of the side trajectory. The coordinates of a trajectory starting at one of the given vertices is given by (see also Fig S9)

(S10) $$x = \int_0^L \cos(\phi(s))\, ds$$

(S11) $$y = \int_0^L \sin(\phi(s))\, ds$$

We assume that to estimate the location of the missing vertex, participants use an average of the two trajectories from the given vertices, and this leads to an expression for the missing vertex coordinates given by

(S12) $$X = \frac{x_L - x_R}{2}\cos(\theta_0) + \frac{y_L - y_R}{2}\sin(\theta_0)$$

(S13) $$Y = \frac{x_L + x_R}{2}\sin(\theta_0) + \frac{y_L + y_R}{2}\cos(\theta_0)$$

Note that x_L, y_L and x_R, y_R are independent and have the same statistics and so we denote $\langle x_L \rangle = \langle x_R \rangle = \langle x \rangle$ and similarly, $\langle y_L \rangle = \langle y_R \rangle = \langle y \rangle$. We also note that by symmetry all odd moments of y will vanish.

Thus, for the moments of X and Y we have

(S14) $$\langle X \rangle = 0$$

(S15) $$\langle Y \rangle = \langle x \rangle \sin\theta_0$$

(S16) $$\langle X^2 \rangle = \frac{1}{2}(\langle x^2 \rangle - \langle x \rangle^2)\cos^2\theta_0 + \frac{1}{2}\langle y^2 \rangle \sin^2\theta_0$$

(S17) $$\langle Y^2 \rangle = \frac{1}{2}(\langle x^2 \rangle - \langle x \rangle^2)\sin^2\theta_0 + \frac{1}{2}\langle y^2 \rangle \cos^2\theta_0$$

and the variances in the position of the missing vertex are given by:

(S18) $$\sigma_X^2 = \frac{1}{2}\sigma_x^2 \cos^2\theta_0 + \frac{1}{2}\sigma_y^2 \sin^2\theta_0$$

(S19) $$\sigma_Y^2 = \frac{1}{2}\sigma_x^2 \sin^2\theta_0 + \frac{1}{2}\sigma_y^2 \cos^2\theta_0$$

Finally, to calculate the skewness in the Y coordinate we need the third moment which is given by:

(S20) $$\langle Y^3 \rangle = \frac{1}{4}(\langle x^3 \rangle + 3\langle x \rangle \langle x^2 \rangle)\sin^3\theta_0 + \frac{3}{4}(\langle xy^2 \rangle + \langle x \rangle \langle y^2 \rangle)\sin\theta_0 \cos^2\theta_0$$

<u>First moment of the x-coordinate</u>:

(S21) $$\langle x \rangle = \int_0^L ds\, \langle \cos\phi(s) \rangle = \int_0^L ds \int_{-\infty}^{\infty} d\phi(s) \cos\phi(s)\, P(\phi(s))$$

(S22) $$P[\phi] \propto \exp\left[-\frac{1}{2}\left(l_p \int_0^L \left(\frac{d\phi}{ds}\right)^2 ds + f\int_0^L (\phi - \theta_0)^2 ds\right)\right]$$

The Gaussian nature of the probability function allows for calculations of the moments of each coordinate estimates using the results for the mean angle and the correlation function

(S23) $\hat{\phi}(s) = \phi_0 e^{-s/\xi}$

(S24) $C(s,s') = \langle \phi(s)\phi(s')\rangle = V_0^2 (e^{-\frac{|s-s'|}{\xi}} - e^{-\frac{s+s'}{\xi}})$

where V_0^2 the variance at each interior angle estimate equals $V_0^2 = 1/\sqrt{4l_p f}$ and $\xi = \sqrt{l_p/f}$ is the correlation length. The probability distribution for each angle along the line is given by

(S25) $P[\phi(s)] = \frac{1}{\sqrt{2\pi C(s,s)}} \exp\left[-\frac{\left(\phi(s)-\hat{\phi}(s)\right)^2}{2C(s,s)}\right]$

and therefore, we can calculate the integrand in (S21) conditioned on the initial angle, as the real part of a complex potential,

(S26) $\langle \cos\phi(s) \mid \phi_0\rangle = \mathrm{Re}\left\{\int_{-\infty}^{\infty} d\phi(s) \frac{1}{\sqrt{2\pi\, C(s,s)}} \exp\left[i\phi(s) - \frac{\left(\phi(s)-\hat{\phi}(s)\right)^2}{2C(s,s)}\right]\right\}$

which yields the following

(S27) $< \cos\phi(s) \,|\phi_0 > = \mathrm{Re}\left\{\exp\left[i\,\hat{\phi}(s) - \frac{1}{2}C(s,s)\right]\right\} =$

$$= \mathrm{Re}\left\{\exp\left[-\frac{1}{2}V_0^2 e^{-\frac{2s}{\xi}} - \frac{1}{2}C(s,s)\right]\right\}$$

Next, we assume a Gaussian distribution for the angle of the first segment

(S28) $P(\phi_0) = \frac{1}{\sqrt{2\pi\sigma_0^2}} \exp\left[-\frac{\phi_0^2}{2\sigma_0^2}\right]$

and on substituting into (S21), we get

(S29) $\langle x\rangle = e^{-\frac{1}{2}V_0^2} \int_0^L ds \exp\left[\frac{1}{2}(V_0^2 - \sigma_0^2)e^{-\frac{2s}{\xi}}\right].$

Using a change in variable with $t = \frac{1}{2}(V_0^2 - \sigma_0^2)e^{-\frac{2s}{\xi}}, dt = -\frac{2}{\xi}t\, ds$ this yields

(S30) $\langle x\rangle = \frac{1}{2}\xi e^{-\frac{1}{2}V_0^2}\left(Ei\left[\frac{V_0^2-\sigma_0^2}{2}\right] - Ei\left[\frac{V_0^2-\sigma_0^2}{2}e^{-2\frac{L}{\xi}}\right]\right)$

where $Ei[x] = \int_{-x}^{\infty} \frac{e^{-t}}{t} dt$ is the exponential integral.

<u>The second moment of both coordinates:</u>

The moments of $\langle x^2\rangle$ and $\langle y^2\rangle$ are

(S31) $\langle x^2\rangle = \int_0^L ds \int_0^L ds' \langle \cos\phi(s)\cos\phi(s')\rangle$

(S32) $\langle y^2\rangle = \int_0^L ds \int_0^L ds' \langle \sin\phi(s)\sin\phi(s')\rangle$

Using the trigonometric identities $2\cos\phi_1\cos\phi_2 = \cos(\phi_1-\phi_2) + \cos(\phi_1+\phi_2) \equiv R + D$ and $2\sin\phi_1\sin\phi_2 = \cos(\phi_1-\phi_2) - \cos(\phi_1+\phi_2) \equiv R - D$ it follows that we need to calculate integrals of the form: $\int_0^L ds \int_0^L ds' \langle \cos(\phi(s) \pm \phi(s'))\rangle =$
$Re\left\{\int_0^L ds \int_0^L ds' \,\langle e^{i\,(\phi(s)\pm\phi(s'))}\rangle\right\}$.

Letting $\vec{\phi} = (\phi(s), \phi(s'))$, the arguments of the exponential can be written as $i\,\vec{k}\,\vec{\phi}$ where $\vec{k} = (1, \pm 1)$. Since the probability distribution (S25) is Gaussian, the above integral calculations are

equivalent to the Fourier transform of a Gaussian with mean $\vec{\hat{\phi}}$ and covariance matrix Σ given by

(S33) $$\vec{\hat{\phi}} = \begin{pmatrix} \hat{\phi}(s) \\ \hat{\phi}(s') \end{pmatrix}, \quad \Sigma = \begin{pmatrix} C(s,s) & C(s,s') \\ C(s',s) & C(s',s') \end{pmatrix}$$

Hence

(S34) $$\langle e^{i\vec{k}\,\vec{\phi}} \rangle = \exp[-\tfrac{1}{2}\, k^T\left(\Sigma + V_0^2 \hat{K}\hat{K}^T\right)k]$$

where $\hat{K}$ is defined by

(S35) $$\hat{\phi} = \phi_0 \begin{pmatrix} e^{-\frac{s}{\xi}} \\ e^{-\frac{s'}{\xi}} \end{pmatrix} \equiv \phi_0 \hat{K}.$$

Thus for R and D we have

(S36) $$R = \int_0^L ds \int_0^L ds' \langle \cos\left(\phi(s) - \phi(s')\right) \rangle =$$
$$= \int_0^L ds \int_0^L ds' \exp\left[-V_0^2\left(1 + e^{-\frac{|s-s'|}{\xi}}\right) + (V_0^2 - \sigma_0^2) e^{-\frac{s+s'}{\xi}}\left(\cosh\left[\frac{s-s'}{\xi}\right] - 1\right)\right]$$

(S37) $$D = \int_0^L ds \int_0^L ds' \langle \cos\left(\phi(s) + \phi(s')\right) \rangle =$$
$$= \int_0^L ds \int_0^L ds' \exp\,[-V_0^2\left(1 + e^{-\frac{|s-s'|}{\xi}}\right) + (V_0^2 - \sigma_0^2) e^{-\frac{s+s'}{\xi}}(\cosh\left[\frac{s-s'}{\xi}\right] + 1)]$$

Making the following change of variables

(S38) $$\begin{pmatrix} t \\ t' \end{pmatrix} = \frac{1}{\xi}\begin{pmatrix} 1 & -1 \\ 1/2 & 1/2 \end{pmatrix}\begin{pmatrix} s \\ s' \end{pmatrix}$$

we get

(S39) $$R = 2\xi^2 \int dt\, e^{-V_0^2(1-e^{-t})}(\mathrm{Ei}[(V_0^2 - \sigma_0^2)(\cosh t - 1)] -$$
$$-\mathrm{Ei}\left[(V_0^2 - \sigma_0^2)(\cosh t - 1)\, e^{t-2\frac{L}{\xi}}\right])$$

(S40) $$D = 2\xi^2 \int dt\, e^{-V_0^2(1+e^{-t})}(Ei[(V_0^2 - \sigma_0^2)(\cosh t + 1)e^{-t}] -$$
$$-Ei\left[(V_0^2 - \sigma_0^2)(\cosh t + 1)e^{t-2\frac{L}{\xi}}\right])$$

Finally, the resulting second moments can be written as:

(S41) $$\langle x^2 \rangle = \frac{1}{2}(R + D)$$

(S42) $$\langle y^2 \rangle = \frac{1}{2}(R - D)$$

<u>The third moment:</u>

The integrals to be computed are

(S43) $\langle x^3\rangle = \int_0^L ds \int_0^L dt \int_0^L du \, \langle \cos\phi(s)\cos\phi(t)\cos\phi(u)\rangle$

(S44) $\langle xy^2\rangle = \int_0^L ds \int_0^L dt \int_0^L du \, \langle \cos\phi(s)\sin\phi(t)\sin\phi(u)\rangle$

Using trigonometric identities yields the following result:

(S45) $\langle x^3\rangle = \frac{1}{4}(M_1 + 3M_2)$

(S46) $\langle xy^2\rangle = \frac{1}{4}(M_2 - M_1)$

where M_1 and M_2 are

(S47) $M_1 = Re\left\{\int_0^L ds \int_0^L dt \int_0^L du \, \langle e^{i\,(\phi(s)+\phi(t)+\phi(u))}\rangle\right\}$

(S48) $M_2 = Re\left\{\int_0^L ds \int_0^L dt \int_0^L du \, \langle e^{i\,(\phi(s)+\phi(t)-\phi(u))}\rangle\right\}$

and we can use again eq. (S34) with the following mean $\hat{\vec{\phi}}$ and covariance matrix Σ

(S49) $\hat{\vec{\phi}} = \phi_0 \begin{pmatrix} e^{-\frac{s}{\xi}} \\ e^{-\frac{t}{\xi}} \\ e^{-\frac{u}{\xi}} \end{pmatrix} \equiv \phi_0 \hat{K}, \; \Sigma = \begin{pmatrix} C(s,s) & C(s,t) & C(s,u) \\ C(t,s) & C(t,t) & C(t,u) \\ C(u,s) & C(u,t) & C(u,u) \end{pmatrix}$

which results in the following equations for M_1 and M_2

(S50) $M_1 = \int_0^L ds \int_0^L dt \int_0^L du \exp\left[-\frac{1}{2}V_0^2\left(3 + 2e^{-\frac{|s-t|}{\xi}} + 2e^{-\frac{|s-u|}{\xi}} + 2e^{-\frac{|t-u|}{\xi}}\right) + \right.$

$\left. + \frac{1}{2}(V_0^2 - \sigma_0^2)e^{-2\frac{s+t+u}{\xi}}\left(e^{\frac{s+t}{\xi}} + e^{\frac{s+u}{\xi}} + e^{\frac{t+u}{\xi}}\right)^2\right]$

(S51) $M_2 = \int_0^L ds \int_0^L dt \int_0^L du \exp\left[-\frac{1}{2}V_0^2\left(3 + 2e^{-\frac{|s-t|}{\xi}} - 2e^{-\frac{|s-u|}{\xi}} - 2e^{-\frac{|t-u|}{\xi}}\right) + \right.$

$\left. + \frac{1}{2}(V_0^2 - \sigma_0^2)e^{-2\frac{s+t+u}{\xi}}\left(e^{\frac{s+t}{\xi}} - e^{\frac{s+u}{\xi}} - e^{\frac{t+u}{\xi}}\right)^2\right]$

S7.3 Calculation of the predicted moments with a noisy base angle

Since we calculated all the moments in the triangle side frame, noisy estimates of base-angles would affect only the rotational part of our calculation. Thus, terms of the type $\sin\theta_0$, $\cos\theta_0$ and their higher powers (see Eqn (S15-20)) will be replaced by their marginalized values under a Gaussian distribution of $\theta_0 \in [0, \pi/2]$

(S52) $P[\theta] = \frac{1}{N(\theta_0,\sigma_\theta)} \exp\left[-\frac{(\theta-\theta_0)^2}{2\sigma_\theta^2}\right], \; \theta \in [0, \pi/2]$

(S53) $N(\theta_0, \sigma_\theta) = \sqrt{\frac{\pi}{2}}\,\sigma_\theta\left(\mathrm{erf}\left[\frac{\frac{\pi}{2}-\theta_0}{\sqrt{2}\sigma_\theta}\right] + \mathrm{erf}\left[\frac{\theta_0}{\sqrt{2}\sigma_\theta}\right]\right)$

and the resulting averaged quantities are:

(S54) $\langle \sin\theta\rangle = e^{-\frac{1}{2}\sigma_\theta^2}\,\mathrm{Im}\left[e^{i\theta_0}\frac{\mathrm{erf}\left[\frac{\frac{\pi}{2}-\theta_0-i\sigma_\theta^2}{\sqrt{2}\sigma_\theta}\right]+\mathrm{erf}\left[\frac{\theta_0+i\sigma_\theta^2}{\sqrt{2}\sigma_\theta}\right]}{\mathrm{erf}\left[\frac{\frac{\pi}{2}-\theta_0}{\sqrt{2}\sigma_\theta}\right]+\mathrm{erf}\left[\frac{\theta_0}{\sqrt{2}\sigma_\theta}\right]}\right]$

(S55) $\langle \sin^2\theta\rangle = \frac{1}{2} - \frac{1}{2}e^{-2\sigma_\theta^2}\,\mathrm{Re}\left[e^{2i\theta_0}\frac{\mathrm{erf}\left[\frac{\frac{\pi}{2}-\theta_0-2i\sigma_\theta^2}{\sqrt{2}\sigma_\theta}\right]+\mathrm{erf}\left[\frac{\theta_0+2i\sigma_\theta^2}{\sqrt{2}\sigma_\theta}\right]}{\mathrm{erf}\left[\frac{\frac{\pi}{2}-\theta_0}{\sqrt{2}\sigma_\theta}\right]+\mathrm{erf}\left[\frac{\theta_0}{\sqrt{2}\sigma_\theta}\right]}\right]$

(S56) $\langle \sin^3\theta\rangle =$

$$\frac{1}{4}\mathrm{Im}\left[\frac{3e^{i\theta_0-\frac{1}{2}\sigma_\theta^2}\left(\mathrm{erf}\left[\frac{\frac{\pi}{2}-\theta_0-i\sigma_\theta^2}{\sqrt{2}\sigma_\theta}\right]+\mathrm{erf}\left[\frac{\theta_0+i\sigma_\theta^2}{\sqrt{2}\sigma_\theta}\right]\right)-e^{3i\theta_0-\frac{9}{2}\sigma_\theta^2}\left(\mathrm{erf}\left[\frac{\frac{\pi}{2}-\theta_0-3i\sigma_\theta^2}{\sqrt{2}\sigma_\theta}\right]+\mathrm{erf}\left[\frac{\theta_0+3i\sigma_\theta^2}{\sqrt{2}\sigma_\theta}\right]\right)}{\mathrm{erf}\left[\frac{\frac{\pi}{2}-\theta_0}{\sqrt{2}\sigma_\theta}\right]+\mathrm{erf}\left[\frac{\theta_0}{\sqrt{2}\sigma_\theta}\right]}\right]$$

and we can calculate the remaining terms by using the relations

(S57) $\langle \cos^2\theta\rangle = 1 - \langle \sin^2\theta\rangle$

(S58) $\langle \sin\theta\cos^2\theta\rangle = \langle sin\theta\rangle - \langle \sin^3\theta\rangle$

S8 Sensitivity analysis of statistical model's moments to parameter variations

The three moments of the location of the missing vertex (see Eqn (S30,S39-40,S50-51) depend on three parameters: the scaled correlation length, ξ/L; the variance of the interior angles, V_o^2; and the variance in the initial angle, σ_0^2.

We first calculated the dependence of the scaling exponent, n ($\sigma\sim L^n$), on the scaled correlation length ξ/L, by varying ξ while keeping other parameters fixed ($L \in [1{,}100], V_0 = 0.26, \sigma_0 = 0$). We found that the exponent increased non-linearly with the correlation length, ξ, from a value of 0.5 towards values above 1, as expected from the scaling analysis (Fig S10A, and see section S7.1). Changing the variance of the interior angles estimates, V_o^2, affects the exponent only slightly, decreasing from 0.77 for low variance to values of 0.6 for high variance levels (up to 120 degrees standard deviation, see Fig S10B). Similarly, keeping length range, interior angles variance and correlation length fixed ($L \in [1{,}100], V_0 = 0.26, \xi = 2$), and varying the initial angle variance, σ_0^2, resulted in a decrease of the scaling exponent (see Fig. S10C). This suggests that the larger the variance, the greater is the impact of the error-correction mechanism. This might correspond to the differences observed in the scaling exponent between the lab and the online experiments, where the latter showed smaller exponents (see section S4).

We then repeated the same analysis for the dependence of the mean deviation, δ, on side length. To do so, we calculated the slope of the mean deviation vs. side length, $\frac{d\delta}{dL}$. We found that the correlation length, ξ, did not significantly affect the slope across two orders of magnitude (Fig. S11A). In contrast, changing the interior angles variance, V_0^2, decreased the slope, making it more and more negative as V_0^2 increases (Fig. S11B). Changing the initial angle variance, σ_0^2, did not affect greatly the resulting deviation slope. However, changing the

variance of base-angle estimates, σ_θ^2, affects the resulting slope mildly, as expected theoretically (Fig. S11C and see section S7).

S9 Fitting experimental estimates in the localization task of the missing vertex to a gamma function distribution

To predict the angle estimates in the angle estimation task and the categorical responses in the verbal geometric reasoning task, we used a Gamma distribution ($\Gamma(x; \alpha, \beta) = \frac{1}{\Gamma(\alpha)\beta^\alpha} x^{\alpha-1} e^{-x/\beta}$), with its two parameters (α=shape, β=scale) taken from the predicted mean and variance values from our model. This allows us to sample from an entire distribution rather than be limited to only its moments. The mapping between the Gamma distribution parameters and the mean and variance values predicted by the model are given by

(S59) $$\alpha = \frac{E[Y]^2}{\mathrm{Var}[Y]}, \quad \beta = \frac{\mathrm{Var}[Y]}{E[Y]}$$

To validate this approach, we used the y-coordinate estimates from the localization task, taking for each triangle its mean and variance and plotting the resulting Gamma distribution compared with the real distribution of participants' responses (Fig S12). We find that the Gamma distribution fits the experimental results of the localization task estimates for most of the triangles presented.

S10 The model predicts the observed variations in the estimates of the missing angle: overestimate increases and variability decreases as side length increases

We first consider the prediction of the missing angle by the dynamical model. In Fig S13, we see that the model predicts an increase in the over-estimate of the angle as triangle size increases, while the variance in angle estimates decreases (compare with Fig S14). We simulated the dynamical process for three base angles=30 degrees, 45 degrees, 60 degrees. The simulation parameters were: integration time scale, τ=1, error-correction time scale, $\xi = 4$, speed $v_p = 1$, base length range, $B \in [10^{0.5}, 10^{2.5}]$, interior angles noise levels, $D = 0.1$, and stopping criterion threshold, $\epsilon = 0.2\, L$.

To estimate the missing angle in the statistical approach we took two approaches. In the first, we used the averaged estimated location to infer the missing angle (missing angle=angle between the two extrapolated sides). In the second, we estimated the right base angle with one estimated location (x_1,y_1) derived from our model and the left base angle with another estimated location (x_2,y_2) (missing angle=180 - effective right base angle - effective left base angle). The two approaches account for two different ways participants might estimate the missing angle – one necessitates holding two lines in visual memory to estimate the angle

between them and the other works sequentially, but necessitates holding the value of the base angles in memory.

Estimates from both methods in the statistical model overestimate the missing angle, and that overestimation increases with a side length increase. In addition, variability in angle estimates decreases as side length increases (Fig. S14A,B). Intuitively, angles overestimates are a manifestation of the vertical bias toward the triangle base, since a lower location of the missing vertex implies smaller 'effective' base angles. The main difference between the two approaches appears in the variance, where in the simulation of the two lines intersection using an averaged location estimate, variance curves for higher base-angles values are smaller. This effect is masked in the estimate of two separate base angles with two different locations.
To test the model's predictions, we asked participants (mechanical Turk, N=65) to estimate the missing angle by moving a virtual goniometer to reflect the angle size (Fig. S14C). We found that as side length increases, participants' angle estimates increase for base angles of 45 and 60 degrees (Fig. S14D). For 30 degrees, the errors in the estimates are smaller and without a clear trend. As predicted by the model, the variability in the estimates decreased as side length increased (Fig. S14D, Inset). We note that in the experimental data, angle estimates show increasing magnitudes of errors for larger base angles, suggesting the involvement of other noise mechanisms that do not result from location estimates. One such example is the noise of the angle estimate itself which calls for further investigation.

S11 Response times of participants in the missing angle task correlate with triangle's size

We analyzed the response times of participants as a function of triangle size in the missing angle estimation task. We find that the mean response time isn't significantly correlated with triangle's side length (Spearman correlation r=0.33, p-value=0.24, Fig 15A). However, when analyzing all response times (not averaged across all participants) we find a significant weak correlation with triangle's side length (Spearman correlation r=0.07, p-value<0.03, Fig 15B). Comparing the two smallest triangle sizes with the two biggest triangle sizes yields a significant difference between the response times (Median(big triangles)-Median(small triangles)=1.4s, d=0.32, Mann-Whitney test: t(16,900)=7048, effect-size=0.17). The correlation of response times with triangle's side length is thus scale dependent and precludes the use of a role such as Euclid's proposition 32 to assess the size of the missing angle.

S12 Years of education have no effect on the accuracy of responses in the categorical geometric reasoning task

In the categorical geometric reasoning task, 407 participants answered 8 categorical questions about the effect of triangle manipulations on the location of the missing vertex and the magnitude of the missing angle. Participants also listed their years of education. To test

whether there was a correlation between accuracy and education level of the participants, we bootstrapped the data 1000 times, each time sampling with returns and calculating the correlation in the bootstrapped data. We found no correlation between participants' accuracy rate and their years of education (Spearman correlation median r=0.002, 95% CI=[0,0.28], see Fig S16).

S13 A "trigonometric" null-model for the categorical geometric reasoning task

To estimate the goodness of fit of our model's predictions to the responses in the categorical geometric reasoning task, we chose to compare it with a null model that assumes only noise in base-angle estimates (and thus straight, unperturbed triangle sides once the angle is set). We note that this model is contradicted by the non-linear scaling of the standard deviation with side length; however, it captures a null model of Euclidean geometry with its noise properties. We refer to this model as the "trigonometric model".

To calculate the trigonometric-model predictions, we used a Gaussian distribution around the base angle with a standard deviation of 5 degrees. To estimate this model's predicted responses to the categorical questions, we calculated the probability that the location after the manipulation (changing of the distance between or angle size of the base angles) would "move up", "move down", or "stay the same" compared to initial triangle. For both location and angle estimates we chose thresholds of 0.05, as implemented for our model in the main text (see also Methods).

The distribution of base angles for the trigonometric model is given by (see section S7.3)

(S60) $$P(\theta_0) = \frac{1}{N(\theta_0,\sigma_\theta)} \exp\left[-\frac{(\theta-\theta_0)^2}{2\sigma_\theta^2}\right], \quad \theta \in [0,\pi/2]$$

(S61) $$N(\theta_0,\sigma_\theta) = \sqrt{\frac{\pi}{2}}\,\sigma_\theta\left(\operatorname{erf}\left[\frac{\pi/2-\theta_0}{\sqrt{2}\sigma_\theta}\right] + \operatorname{erf}\left[\frac{\theta_0}{\sqrt{2}\sigma_\theta}\right]\right)$$

and therefore, the vertical location estimates distribution is

(S62) $$P(Y) = \frac{1}{N(\theta_0,\sigma_\theta)\sqrt{L^2-Y^2}} \exp\left[-\frac{\left(\sin^{-1}\left[\frac{Y}{L}\right]-\theta_0\right)^2}{2\sigma_\theta^2}\right], \quad \theta \in [0,\pi/2]$$

Define Y_1 as the vertical location estimate of a triangle with side length L_1 (initial triangle) and Y_2 as the vertical location estimate of a triangle with side length L_2, (the triangle after the distance manipulation). We next denote, $Y_\downarrow(Y_1) = 0.95\,Y_1$ and $Y_\uparrow(Y_1) = 1.05\,Y_1$ as the lower and upper boundaries of the 'same' range.

Integrating over the joint probability for Y_1 and Y_2 we can compute the probabilities for "moves down" (marked with a downward arrow) and "moves up" (marked with an upward arrow) responses

(S63) $$P(\downarrow_{loc}\,|H_{trig}) = \int_0^{L_1} dY_1 \int_0^{Y_\downarrow(Y_1)} dY_2\, P(Y_1,Y_2)$$

(S64) $P\left(\uparrow_{loc}\left|H_{trig}\right.\right)=\int_0^{L_1}dY_1\int_{Y_\uparrow(Y_1)}^{L_2}dY_2\,P(Y_1,Y_2)$

Since Y_1 and Y_2 are independent, each given by a different process on a different triangle we can write the probabilities as

(S65) $P\left(\downarrow_{loc}\left|H_{trig}\right.\right)=\int_0^{L_1}dY_1\,P(Y_1)P(\downarrow|Y_1)$

(S66) $P\left(\uparrow_{loc}\left|H_{trig}\right.\right)=\int_0^{L_1}dY_1\,P(Y_1)P(\uparrow|Y_1)$

(S67) $P(\downarrow|Y_1)=\int_0^{Y_\downarrow(Y_1)}dY_2\,P(Y_2)=\dfrac{\mathrm{erf}\left[\frac{\theta_0}{\sqrt{2}\sigma_\theta}\right]-\mathrm{erf}\left[\frac{1}{\sqrt{2}\sigma_\theta}\left(\theta_0-\sin^{-1}\left[\frac{Y_\downarrow(Y_1)}{L_2}\right]\right)\right]}{\mathrm{erf}\left[\frac{\pi/2-\theta_0}{\sqrt{2}\sigma_\theta}\right]+\mathrm{erf}\left[\frac{\theta_0}{\sqrt{2}\sigma_\theta}\right]}$

(S68) $P(\uparrow|Y_1)=\int_{Y_\uparrow(Y_1)}^{L_2}dY_2\,P(Y_2)=\dfrac{\mathrm{erf}\left[\frac{\pi/2-\theta_0}{\sqrt{2}\sigma_\theta}\right]-\mathrm{erf}\left[\frac{1}{\sqrt{2}\sigma_\theta}\left(\sin^{-1}\left[\frac{Y_\uparrow(Y_1)}{L_2}\right]-\theta_0\right)\right]}{\mathrm{erf}\left[\frac{\pi/2-\theta_0}{\sqrt{2}\sigma_\theta}\right]+\mathrm{erf}\left[\frac{\theta_0}{\sqrt{2}\sigma_\theta}\right]}$

which when plugging back to eqn (S65-S66) yields

(S69) $P\left(\downarrow_{loc}\left|H_{trig}\right.\right)=$

$$\frac{\sqrt{2}}{\sqrt{\pi}\sigma_\theta}\int_0^{L_1}dY_1\,\frac{\mathrm{erf}\left[\frac{\theta_0}{\sqrt{2}\sigma_\theta}\right]-\mathrm{erf}\left[\frac{1}{\sqrt{2}\sigma_\theta}\left(\theta_0-\sin^{-1}\left[\frac{Y_\downarrow(Y_1)}{L_2}\right]\right)\right]}{\left(\mathrm{erf}\left[\frac{\frac{\pi}{2}-\theta_0}{\sqrt{2}\sigma_\theta}\right]+\mathrm{erf}\left[\frac{\theta_0}{\sqrt{2}\sigma_\theta}\right]\right)^2\sqrt{L_1^2-Y_1^2}}\exp\left[-\frac{\left(\sin^{-1}\left[\frac{Y_1}{L_1}\right]-\theta_0\right)^2}{2\sigma_\theta^2}\right]$$

(S70) $P\left(\uparrow_{loc}\left|H_{trig}\right.\right)=$

$$\frac{\sqrt{2}}{\sqrt{\pi}\sigma_\theta}\int_0^{L_1}dY_1\,\frac{\mathrm{erf}\left[\frac{\pi/2-\theta_0}{\sqrt{2}\sigma_\theta}\right]-\mathrm{erf}\left[\frac{1}{\sqrt{2}\sigma_\theta}\left(\sin^{-1}\left[\frac{Y_\uparrow(Y_1)}{L_2}\right]-\theta_0\right)\right]}{\left(\mathrm{erf}\left[\frac{\frac{\pi}{2}-\theta_0}{\sqrt{2}\sigma_\theta}\right]+\mathrm{erf}\left[\frac{\theta_0}{\sqrt{2}\sigma_\theta}\right]\right)^2\sqrt{L_1^2-Y_1^2}}\exp\left[-\frac{\left(\sin^{-1}\left[\frac{Y_1}{L_1}\right]-\theta_0\right)^2}{2\sigma_\theta^2}\right]$$

Similarly, for the angle estimates we compared the angles before and after the manipulations, with the same Gaussian distribution for the base angles and the same thresholds. Following Euclidean rules, the triangle length-scale does not play a role in these comparisons

(S71) $P\left(\downarrow_{ang}\left|H_{trig}\right.\right)=\dfrac{\sqrt{2}}{\sqrt{\pi}\sigma_\theta}\int_0^{\pi/2}d\theta_1\,\dfrac{\mathrm{erf}\left[\frac{\theta_0}{\sqrt{2}\sigma_\theta}\right]-\mathrm{erf}\left[\frac{1}{\sqrt{2}\sigma_\theta}(\theta_0-\theta_\downarrow(\theta_1))\right]}{\left(\mathrm{erf}\left[\frac{\frac{\pi}{2}-\theta_0}{\sqrt{2}\sigma_\theta}\right]+\mathrm{erf}\left[\frac{\theta_0}{\sqrt{2}\sigma_\theta}\right]\right)^2}\exp\left[-\dfrac{(\theta_1-\theta_0)^2}{2\sigma_\theta^2}\right]$

(S72) $P\left(\uparrow_{ang}\left|H_{trig}\right.\right)=\dfrac{\sqrt{2}}{\sqrt{\pi}\sigma_\theta}\int_0^{\pi/2}d\theta_1\,\dfrac{\mathrm{erf}\left[\frac{\pi/2-\theta_0}{\sqrt{2}\sigma_\theta}\right]-\mathrm{erf}\left[\frac{1}{\sqrt{2}\sigma_\theta}(\theta_\uparrow(\theta_1)-\theta_0)\right]}{\left(\mathrm{erf}\left[\frac{\frac{\pi}{2}-\theta_0}{\sqrt{2}\sigma_\theta}\right]+\mathrm{erf}\left[\frac{\theta_0}{\sqrt{2}\sigma_\theta}\right]\right)^2}\exp\left[-\dfrac{(\theta_1-\theta_0)^2}{2\sigma_\theta^2}\right]$

For estimates of locations and angles after an angle manipulation, we computed the respective integrals for the initial triangle side-length and base-angle (*L*, $\theta_{0,1}$) and the manipulated triangle parameters (*L*, $\theta_{0,2}$)

(S73) $P\left(\downarrow_{loc}\left|H_{trig}\right.\right)=$

$$\frac{\sqrt{2}}{\sqrt{\pi}\sigma_\theta}\int_0^{L}dY_1\,\frac{\mathrm{erf}\left[\frac{\theta_{0,2}}{\sqrt{2}\sigma_\theta}\right]-\mathrm{erf}\left[\frac{1}{\sqrt{2}\sigma_\theta}\left(\theta_{0,2}-\sin^{-1}\left[\frac{Y_\downarrow(Y_1)}{L}\right]\right)\right]}{\left(\mathrm{erf}\left[\frac{\frac{\pi}{2}-\theta_{0,1}}{\sqrt{2}\sigma_\theta}\right]+\mathrm{erf}\left[\frac{\theta_{0,1}}{\sqrt{2}\sigma_\theta}\right]\right)\left(\mathrm{erf}\left[\frac{\frac{\pi}{2}-\theta_{0,2}}{\sqrt{2}\sigma_\theta}\right]+\mathrm{erf}\left[\frac{\theta_{0,2}}{\sqrt{2}\sigma_\theta}\right]\right)\sqrt{L^2-Y_1^2}}\exp\left[-\frac{\left(\sin^{-1}\left[\frac{Y_1}{L}\right]-\theta_{0,1}\right)^2}{2\sigma_\theta^2}\right]$$

(S74) $$P\left(\uparrow_{loc} \mid H_{trig}\right) =$$

$$\frac{\sqrt{2}}{\sqrt{\pi}\sigma_\theta}\int_0^L dY_1 \frac{\operatorname{erf}\left[\frac{\pi/2-\theta_{0,2}}{\sqrt{2}\sigma_\theta}\right]-\operatorname{erf}\left[\frac{1}{\sqrt{2}\sigma_\theta}\left(\sin^{-1}\left[\frac{Y_\uparrow(Y_1)}{L}\right]-\theta_{0,2}\right)\right]}{\left(\operatorname{erf}\left[\frac{\frac{\pi}{2}-\theta_{0,1}}{\sqrt{2}\sigma_\theta}\right]+\operatorname{erf}\left[\frac{\theta_{0,1}}{\sqrt{2}\sigma_\theta}\right]\right)\left(\operatorname{erf}\left[\frac{\frac{\pi}{2}-\theta_{0,2}}{\sqrt{2}\sigma_\theta}\right]+\operatorname{erf}\left[\frac{\theta_{0,2}}{\sqrt{2}\sigma_\theta}\right]\right)\sqrt{L^2-Y_1^2}} \exp\left[-\frac{\left(\sin^{-1}\left[\frac{Y_1}{L_1}\right]-\theta_{0,1}\right)^2}{2\sigma_\theta^2}\right]$$

(S75) $$P\left(\downarrow_{ang} \mid H_{trig}\right) = \frac{\sqrt{2}}{\sqrt{\pi}\sigma_\theta}\int_0^{\pi/2} d\theta_1 \frac{\operatorname{erf}\left[\frac{\theta_0}{\sqrt{2}\sigma_\theta}\right]-\operatorname{erf}\left[\frac{1}{\sqrt{2}\sigma_\theta}(\theta_0-\theta_\downarrow(\theta_1))\right]}{\left(\operatorname{erf}\left[\frac{\frac{\pi}{2}-\theta_0}{\sqrt{2}\sigma_\theta}\right]+\operatorname{erf}\left[\frac{\theta_0}{\sqrt{2}\sigma_\theta}\right]\right)^2} \exp\left[-\frac{(\theta_1-\theta_0)^2}{2\sigma_\theta^2}\right]$$

(S76) $$P\left(\uparrow_{ang} \mid H_{trig}\right) = \frac{\sqrt{2}}{\sqrt{\pi}\sigma_\theta}\int_0^{\pi/2} d\theta_1 \frac{\operatorname{erf}\left[\frac{\pi/2-\theta_0}{\sqrt{2}\sigma_\theta}\right]-\operatorname{erf}\left[\frac{1}{\sqrt{2}\sigma_\theta}(\theta_\uparrow(\theta_1)-\theta_0)\right]}{\left(\operatorname{erf}\left[\frac{\frac{\pi}{2}-\theta_0}{\sqrt{2}\sigma_\theta}\right]+\operatorname{erf}\left[\frac{\theta_0}{\sqrt{2}\sigma_\theta}\right]\right)^2} \exp\left[-\frac{(\theta_1-\theta_0)^2}{2\sigma_\theta^2}\right]$$

Lastly, the probabilities for the ‘same’ events are calculated by

(S77) $$P\left(= \mid H_{trig}\right) = 1 - P\left(\downarrow \mid H_{trig}\right) - P\left(\uparrow \mid H_{trig}\right)$$

S14 Sensitivity analysis of the model predictions in the categorical geometric reasoning task

To assess the model’s parameter sensitivity for each parameter, we kept other parameters fixed at their given values in the main text while varying the parameter in question. For each parameter, we calculated the resulting probabilities (“gets bigger”, “gets smaller”, “stays the same”) for the four categorical questions – ‘VIA’ (question about the missing vertex location after increasing the base angles), ‘VID’ (question about the missing vertex location after increasing the distance between the base corners), ‘AIA‘ (question about the missing angle size after increasing the base angles) and ‘AID’ (question about the missing angle size after increasing the distance between the base corners). As in the main text, angle changes were from 36 degrees to 45 degrees and length changes incurred an 25% change in the location questions and 50% in the angle questions.

We found that the initial length of the triangle significantly changed the probabilities only in the ‘AID’ condition, with increasing current trends in the other conditions. Increasing the initial side length in the ‘VIA’ and ‘VID’ conditions raised the probability for estimates of the location change as “moves up” at the expense of the probability for estimating a “moves down” (Fig S17A,B). Similarly, the estimate of angles getting “smaller” for increasing base-angles sizes became greater as length increased (Fig. S17C). For the ‘AID’ condition, as length increased the probability of “stays the same” increased while the two probabilities of “gets smaller” and “gets bigger” decrease (Fig. S17D). In conclusion, as expected from the model, as side length, L, increased while the correlation length, ξ, is fixed, the model’s predictions become more and more “Euclidean”. The sensitivity of the categorical probabilities predictions to variations of the

correlation length ξ (Min-Max:0.3-15) behaves in an inverse manner to the effect of length changes, though on a smaller scale (S18A-D).

Next, we checked the dependence of the categorical probabilities on the thresholds values (Th_L, Th_A). We varied the threshold between 5% to 20% and found a gradual dependency of the probabilities on the threshold (Fig. S19A-D). For the condition 'AIA', changing the threshold results in asymmetrical decrease in "bigger" vs. "smaller" probabilities, indicating a skew in the model's resulting distribution for the change in angles with increasing base-angles size (Fig. S19C).

Lastly, increasing the variance of the interior-angles estimates, V_0^2, transitions the model predictions from pure "Euclidean" to worse and worse estimates of the categorical probabilities, indicating an increasing difficulty to assess the change in location and angle size and the increased variance of each of these estimated distributions (Fig. S20A-D).

S15 The statistics of the location of the missing vertex as a function of the base angle

The observed location of the missing vertex had a mean value that scaled linearly with side length. Here, we show that a simple model of noisy estimates of the base angle accounted for the observed relation between the mean location of the missing vertex and the base angle. We start with the simple prediction of the y-coordinate's dependence on the base length ($B = 2L\cos\theta$) and base angle (θ):

(S78) $$y = \frac{B}{2}\tan\theta$$

Assuming a Gaussian estimate of the base angle, $P(\theta) \sim \exp[-\frac{(\theta-\theta_0)^2}{2\sigma_0^2}]$, the y-coordinate distribution is:

(S79) $$P[y] \sim \frac{2}{B\left(1+\left(\frac{2y}{B}\right)^2\right)} \exp[-\frac{1}{2\sigma_0^2}\left(\theta_0 - \arctan[\frac{2y}{B}]\right)^2]$$

and the maximum likelihood is achieved at

(S80) $$\theta_0 = \frac{4\hat{y}}{B}\sigma_0^2 + \arctan\left[\frac{2\hat{y}}{B}\right]$$

In the limit of small noise for a base-angle estimate, $\sigma_0 \ll 1$. Therefore, the vertical location of the missing vertex has a small deviation from its true location which is linearly dependent on the length, i.e. $\hat{y} = \frac{B}{2}(\tan\theta_0 + \delta)$. Substituting this relation into (S80), we get

(S81) $$\theta_0 = 2\,\sigma_0^2(\tan\theta_0 + \delta) + \theta_0 + \frac{\delta}{1+\tan^2\theta_0}$$

which yields

(S82) $$\delta = -\frac{2\,\sigma_0^2\tan\theta_0}{2\,\sigma_0^2+\cos^2\theta_0}$$

so that the mean location of the missing vertex is

(S83) $$\hat{y} = \frac{B}{2}\tan\theta_0\left(1 - \frac{2\,\sigma_0^2}{2\,\sigma_0^2 + \cos^2\theta_0}\right)$$

showing a systematic downward trend for the location, with a prediction for a specific relationship to the base angle. In Figure S21, we show the fit of this prediction with the data from the online experiment, where angles varied in a range between 30-66 degrees. We used a fit of the deviation for large base-lengths folds (0.5, 0.75 and 1) since the noise at lower base lengths was too high to enable a reliable fit. For all three base lengths, there was a good fit between the model predictions and the observed location-estimate deviations in the localization task of the online experiment. We note that parameter estimates showed high variability, indicating a possible need for a more complex model.

Figures

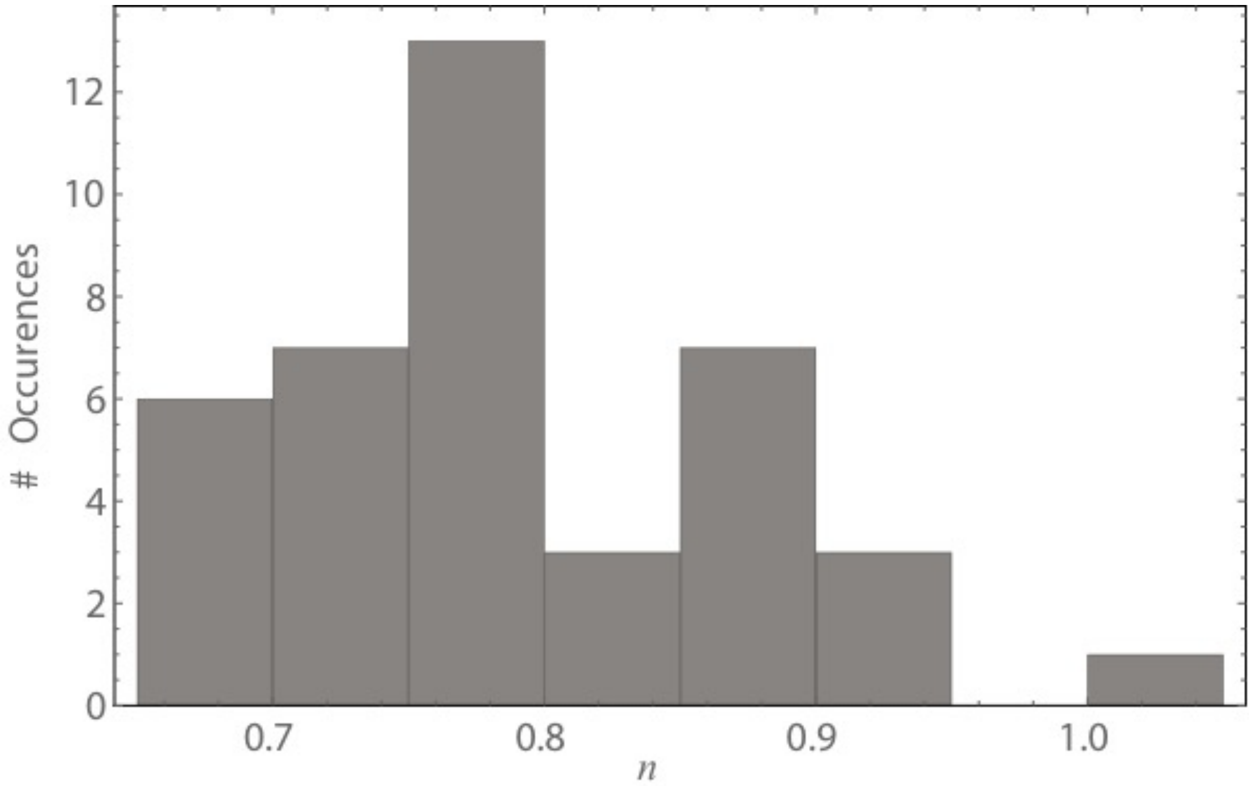

Figure S1: Histogram of y-coordinate scaling exponents for individual participants. Each participant was shown 15 different triangles, composed of 5 base lengths and 3 angles conditions; each triangle was presented 10 times. For each participant, we calculated the scaling exponent, n, of the y-coordinate standard deviation with the side length per participant ($\sigma \sim L^n$).

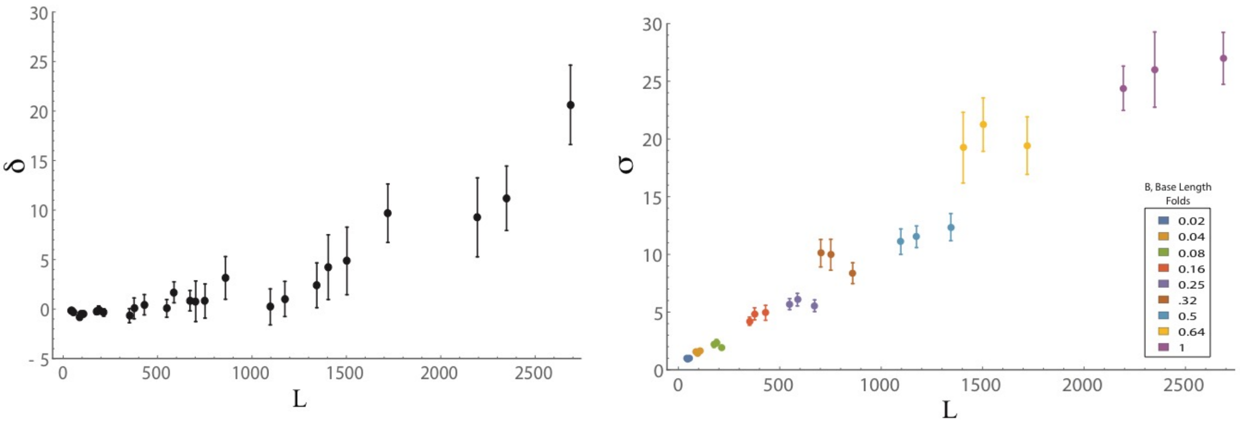

Figure S2: X-coordinate mean deviation from the missing vertex location and standard deviation scaling with triangle side-length. A) The x-coordinate estimates of the missing vertex were close to the true location for most side lengths, increasing with side length for high values only. Shown are values of the mean deviation from the true x location, δ, as side length, L, increases. B) The scaling exponent of the x-coordinate standard deviation with the side-length scales sub-linearly with side length, with a scaling exponent of 0.86 (median exponent=0.86, 95% CI=[0.84,0.87]). Shown are values of the x-coordinate standard deviation, σ, as a function of the side length, L.

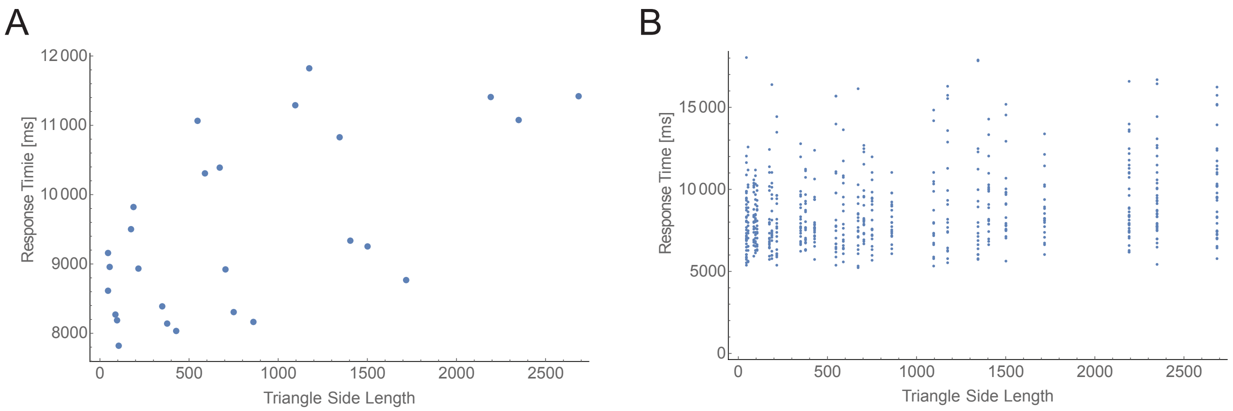

Figure S3: Response times correlate with triangle's side length in the localization task. A) we analyzed the response times of participants as a function of triangle size in the vertex localization task. We find that the mean response time significantly correlates with triangle's side length (Spearman correlation r=0.53, p-value<0.005). **B)** A second analysis with all response times (not averaged across all participants) still shows a correlation with triangle's side length (Spearman correlation r=0.22, p-value$<10^{-7}$).

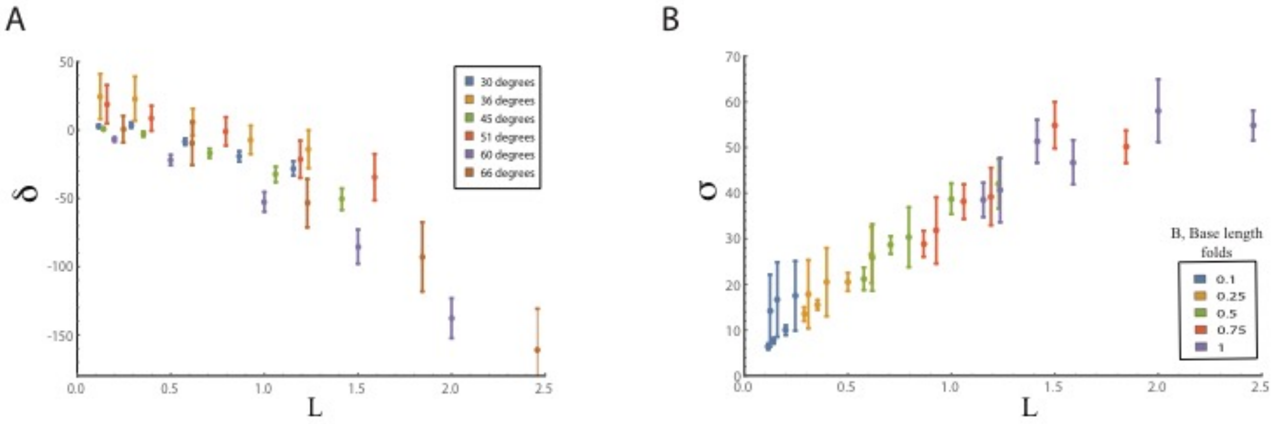

Figure S4: Online experiment results show a bias toward the triangle's base and a sub-linear scaling exponent of y-coordinate standard deviation with side length. 100 participants completed the online study in two experimental setups. All participants were shown 15 triangles, at base lengths of 0.1, 0.25, 0.5, 0.75, 1 of the maximal base length (Group 1 – 900 pixels, Group 2 – 1300 pixels; Group 2 locations were scaled by the ratio of the two experiments to match scale on screen). Each base length was presented with 3 different angles (Group 1 – 30, 45 and 60 degrees, Group 2 - 36, 51 and 66 degrees). Each triangle condition was repeated 10 times, totaling 150 trials. A) The error of the vertical mean estimate, δ, was biased toward the base, increasing linearly with side length, L. B) The y-coordinate standard deviation, σ, scaled sub-linearly with the side length, L, with a mean exponent of 0.64: $\sigma \sim L^{0.64}$. In both panels, side length is normalized by the base length value (*B*) in pixels.

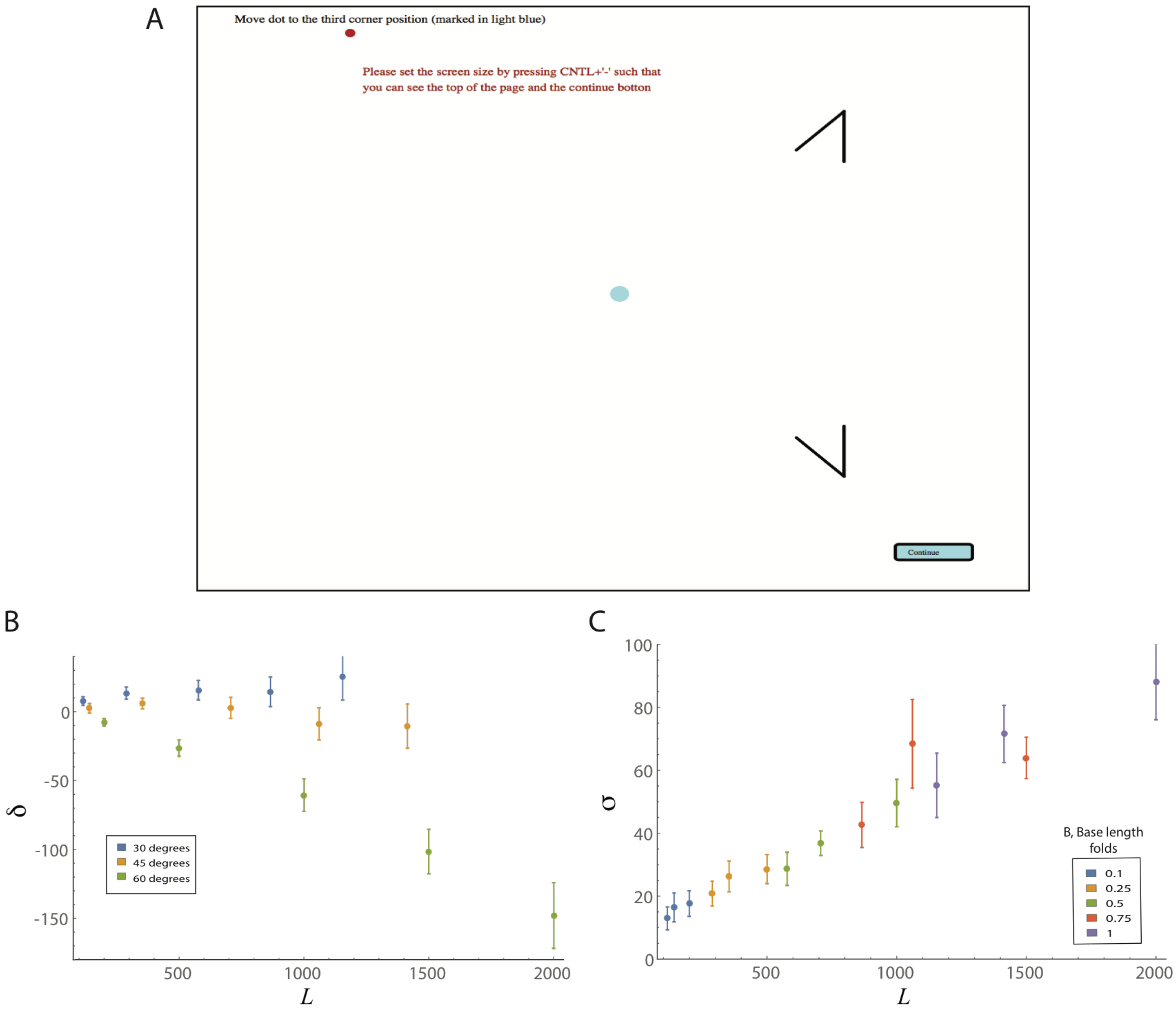

Figure S5: A tilted version of the localization task is biased towards the triangle base and sub-linear scaling of the standard deviation with side-length. A) An illustration of the tilted triangle completion task. The triangle base was at the right side of the screen on the y-axis. **B)** The mean deviation of participants' location estimates in the x-axis, δ as a function of side length, L. For 45 and 60 degrees, the deviation, δ, was biased toward the triangle base and increased with side length. For 30 degrees, the bias was opposite to the base, increasing slightly with side length. **C)** The x-coordinate location estimates standard deviation, σ, scaled sub-linearly with side length, $\sigma \sim L^{0.66}$. It should be noted that noise levels in the experiment were higher than other online experiments, perhaps reflecting participants' difficulty with the task.

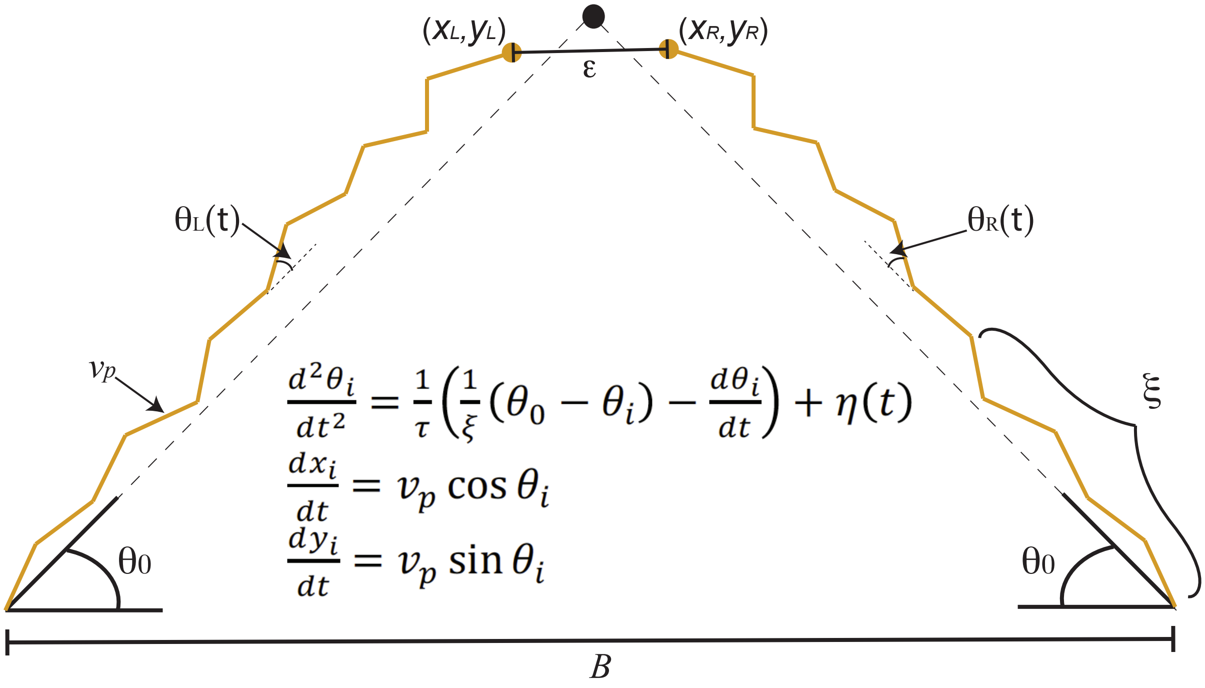

Figure S6: The schematics of the dynamical model parameters in the triangle completion task. The model parameters are: v_p, a characteristic speed with which the coordinates progress, ξ, a time scale for global error-correction (illustrated as number of segments between error-correction events), and $\eta(t)$ is a noise term with noise amplitude D ($\langle\eta(t)\eta(t')\rangle = D\,\delta(t - t')$), not shown in the figure. The base length is denoted as *B,* and the stopping criterion threshold is denoted, ϵ. The base angle is denoted by θ_0.

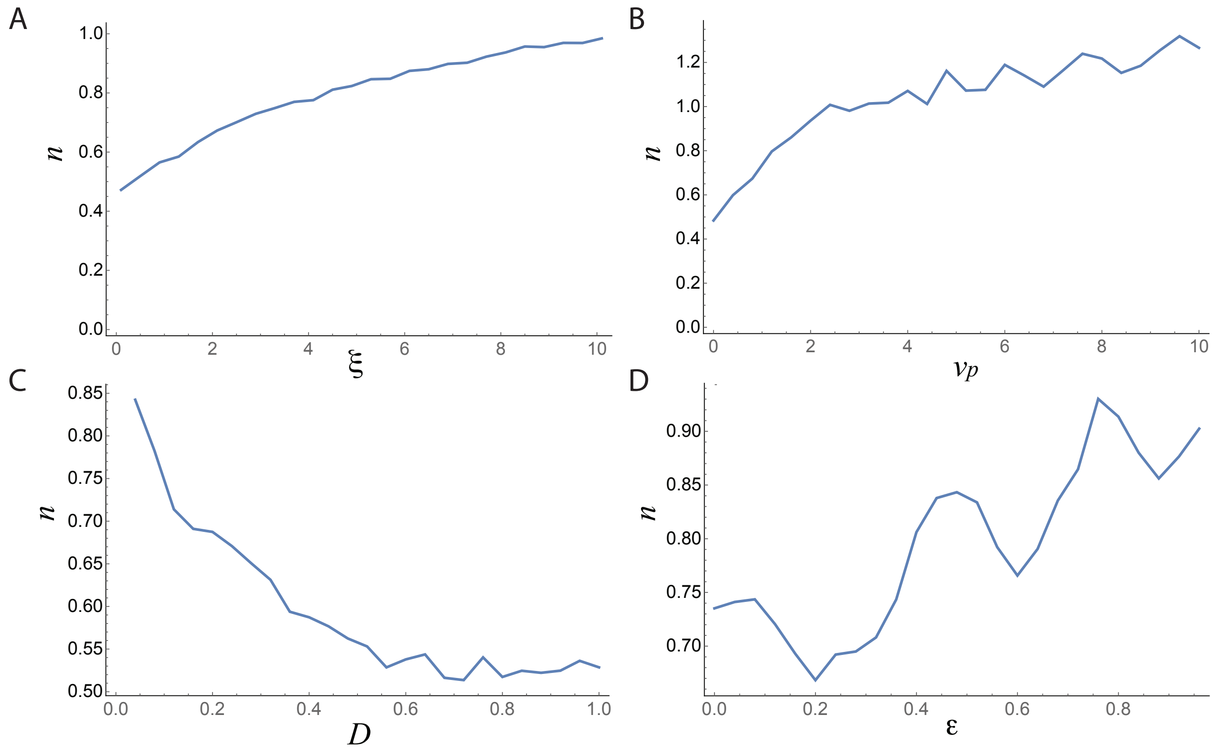

Figure S7: The dynamic model's scaling exponent, n, sensitivity to parameters A) Error-correction time scale, ξ **B)** Characteristic speed, v_p **C)** Interior-angles noise level, D **D)** Stopping criterion, ϵ

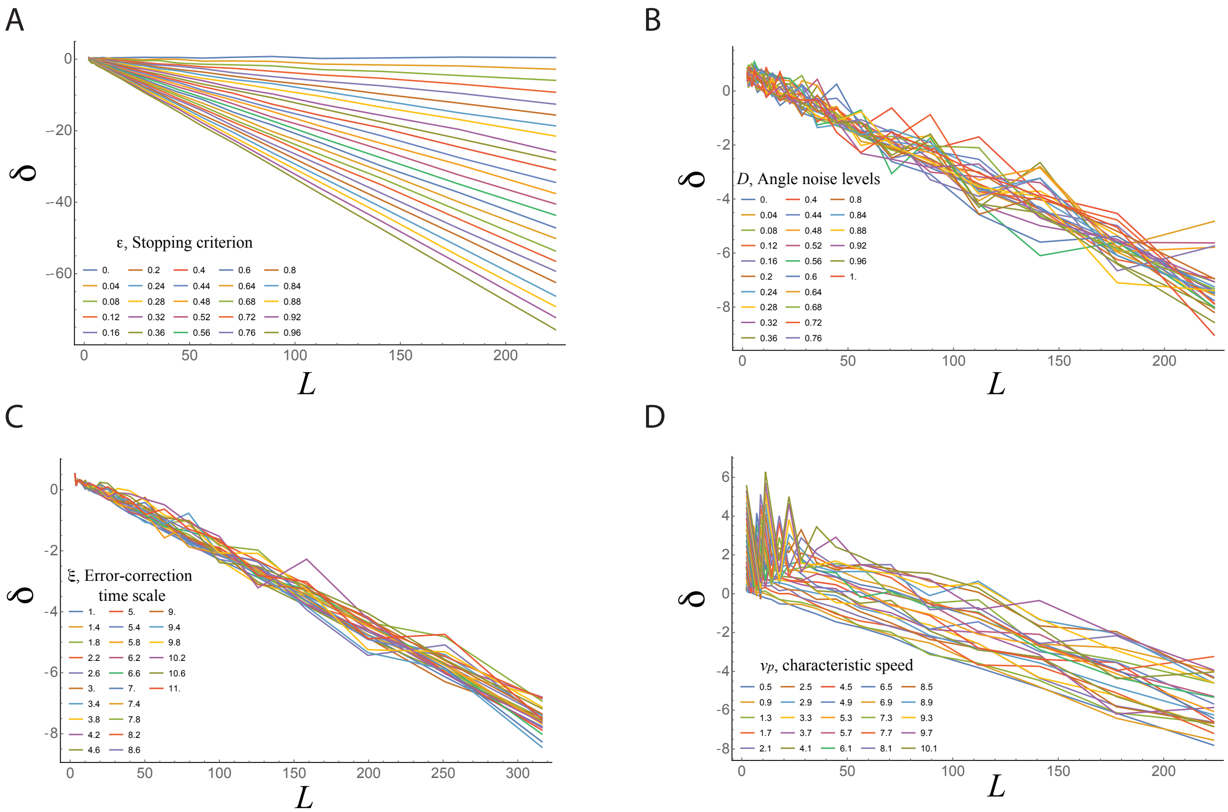

Figure S8: The dynamic model's mean deviation, δ, sensitivity to parameters A) Stopping criterion, ϵ **B) I**nterior-angles noise level, D **C)** Error-correction time scale, ξ **D)** Characteristic speed, v_p

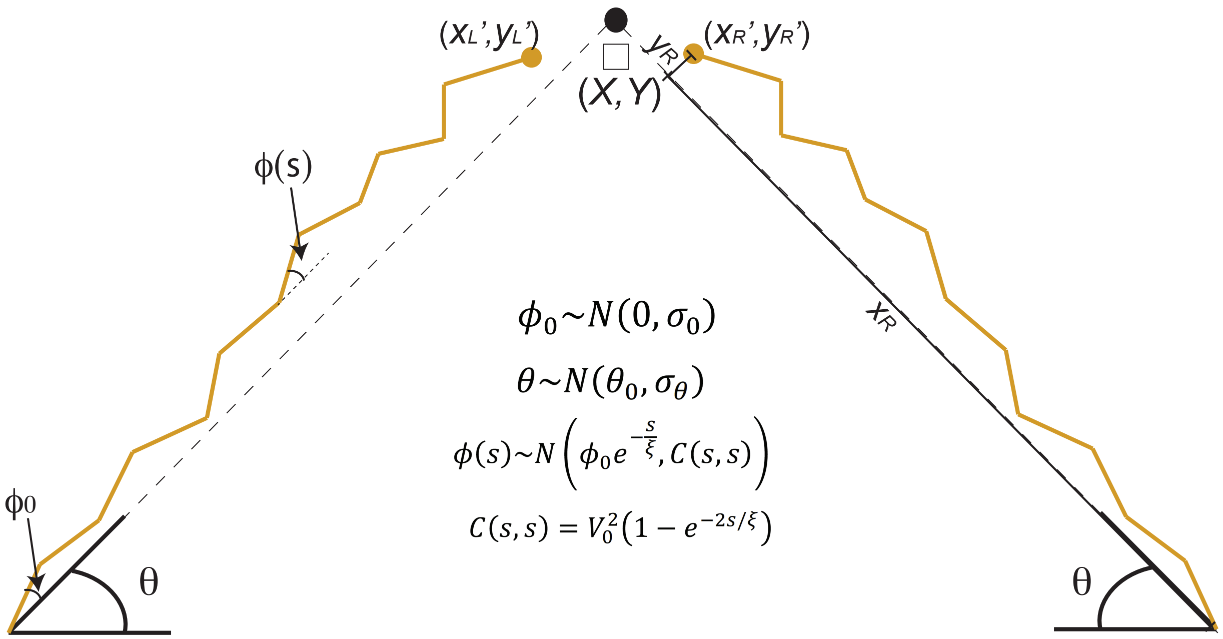

Figure S9: The schematics of the model parameters in the triangle completion task. The model has three different angles – ϕ_0, the initial angle of the extrapolated side curve, $\phi(s)$, the interior angle at a distance s away and the base angle θ. Each angle has a Gaussian distribution with mean and variance. σ_0^2, is the variance of the angle ϕ_0. V_0^2, is the variance amplitude of the interior-angles estimates and σ_θ^2, is the variance of the base angle, centered at θ_0. For the estimated location *(X,Y)* we calculate the statistics of the right side trajectory of the triangle (x_R, y_R) and then rotate it to get the moments for the rotated coordinates $(x_R', y_R') = \begin{pmatrix} \cos\theta & -\sin\theta \\ \sin\theta & \cos\theta \end{pmatrix} \begin{pmatrix} x_R \\ y_R \end{pmatrix}$, and similarly for the left side trajectory of the triangle (x_L', y_L'). The two side trajectories are independent and assumed to have the same statistics.

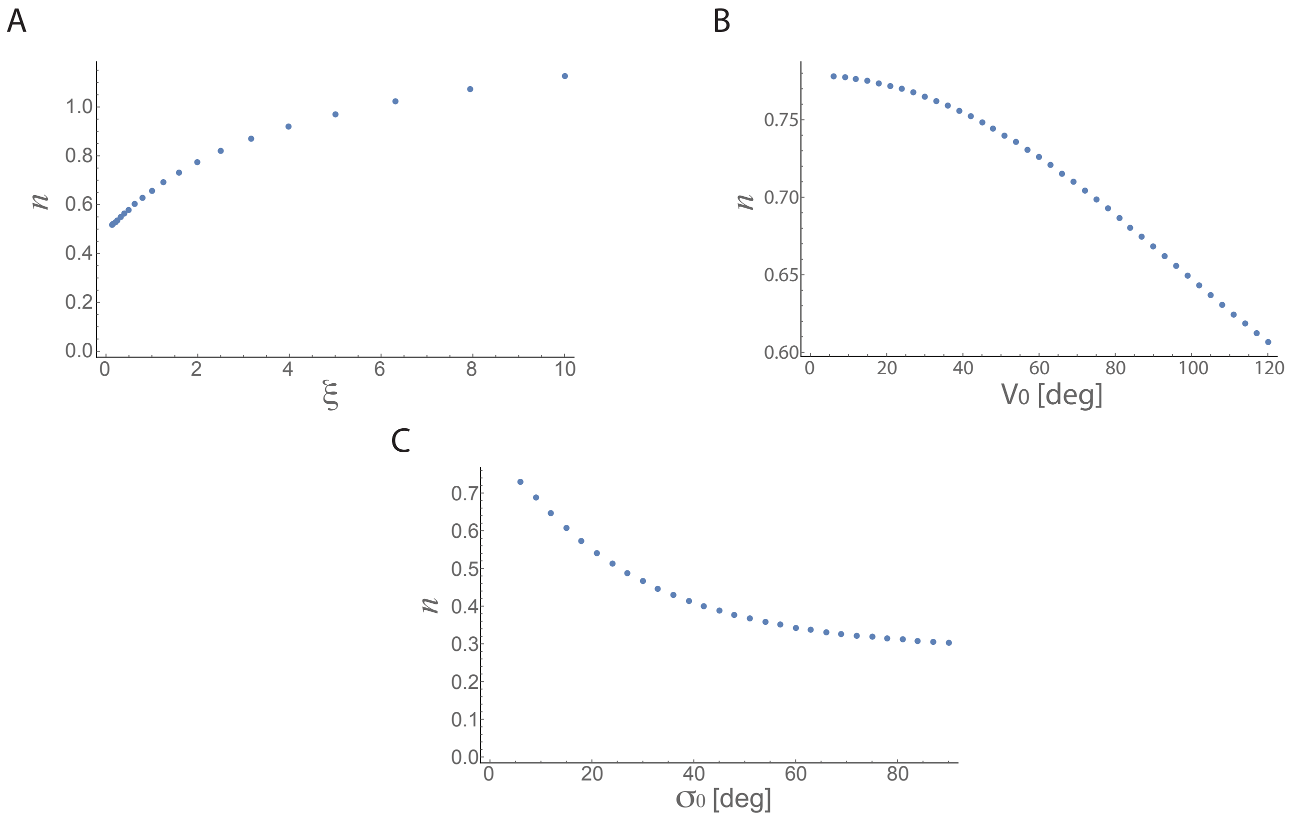

Figure S10: The sensitivity of the scaling exponent, n, to parameters of the statistical model. **A)** Correlation length, ξ **B)** Standard deviation of interior-angles estimates, V_0 **C)** Initial angle standard deviation levels, σ_0

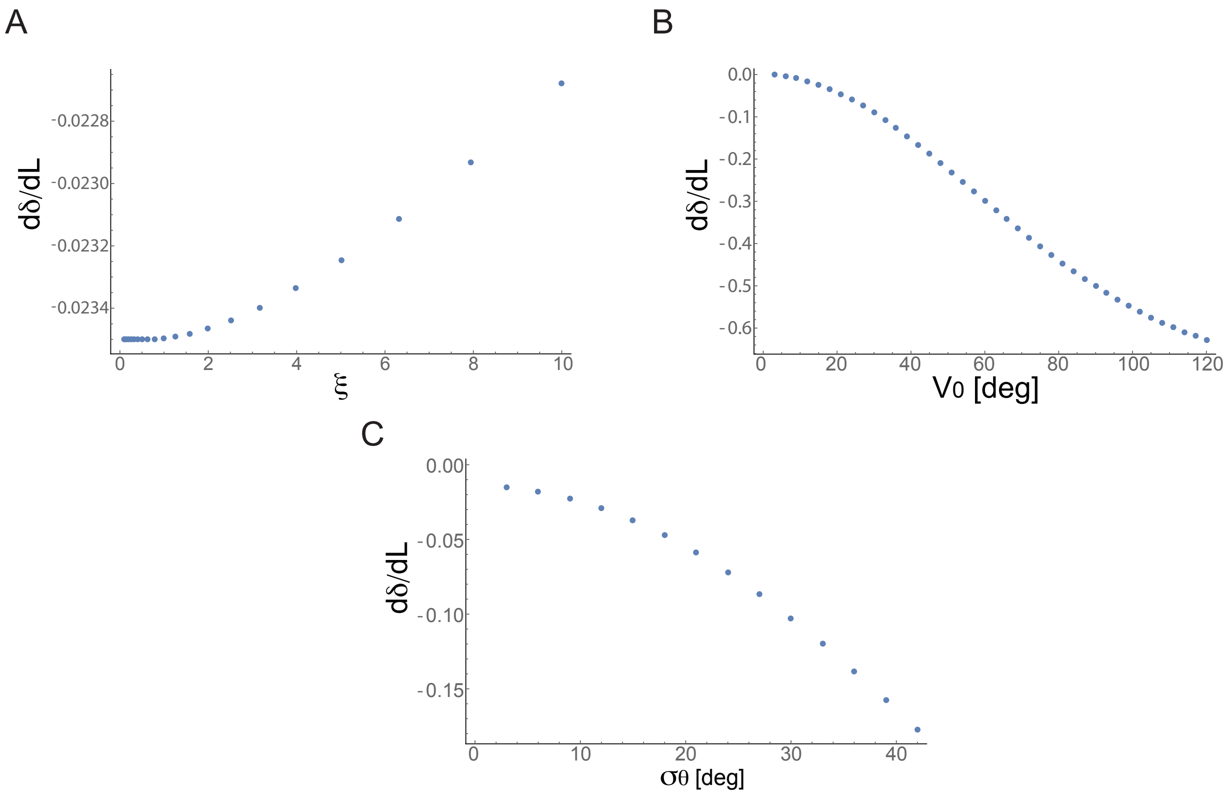

Figure S11: The sensitivity of the model's predictions for the deviation of the vertex location as a function of triangle size, $\frac{d\delta}{dL}$, to parameters A) Correlation length, ξ **B)** Standard deviation of interior-angles estimates, V_0 **C)** Base angle standard deviation, σ_θ

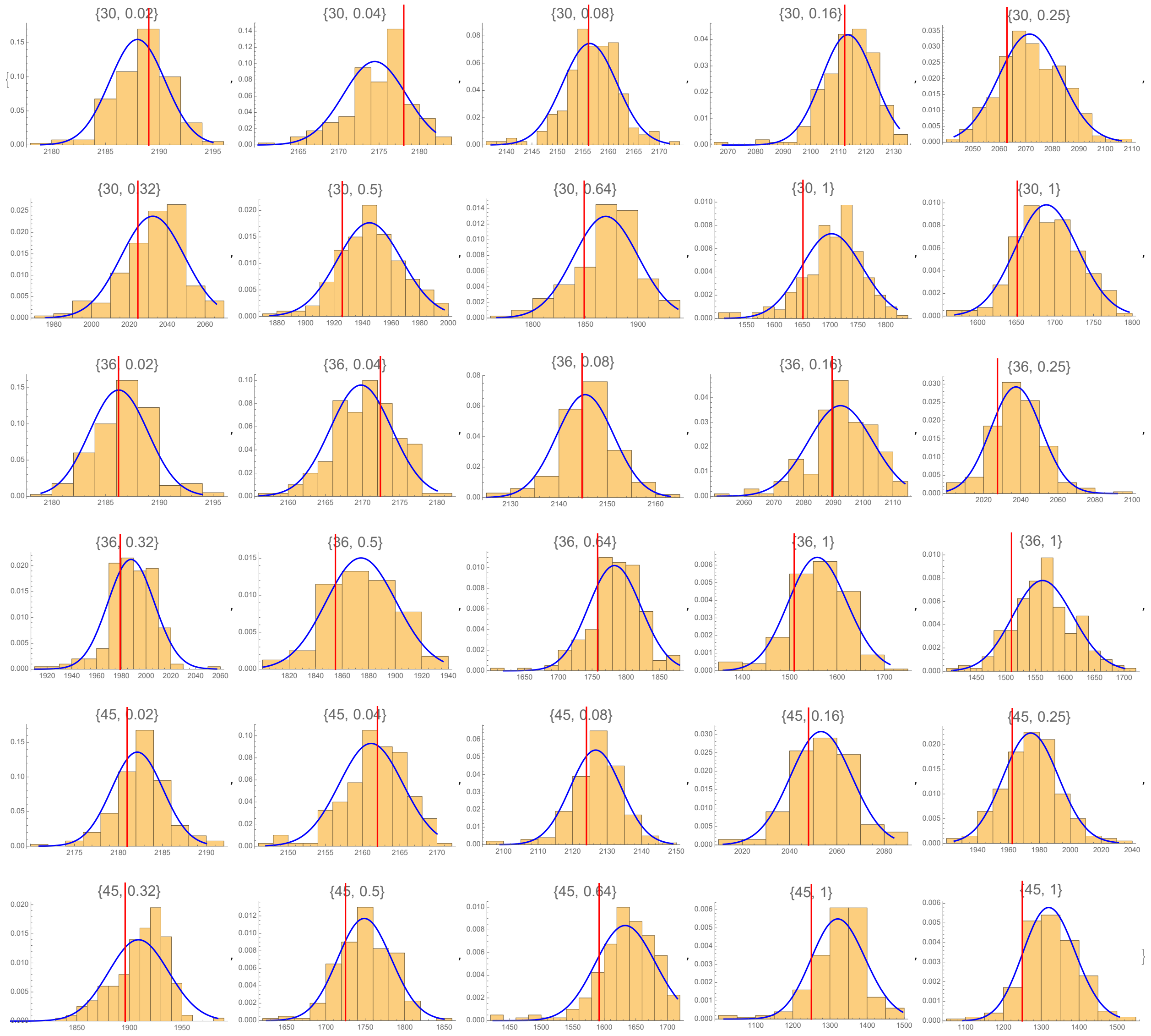

Figure S12: Fitting results of a Gamma distribution, $\Gamma(x;\alpha,\beta)=\frac{1}{\Gamma(\alpha)\beta^{\alpha}}x^{\alpha-1}e^{-x/\beta}$, to the vertical location estimates in the localization task in the lab experiment. Plot labels are angle and base factor of the triangle condition, red line signifies the real y-coordinate value of the missing vertex (the higher the y-value the lower is its position on the screen).

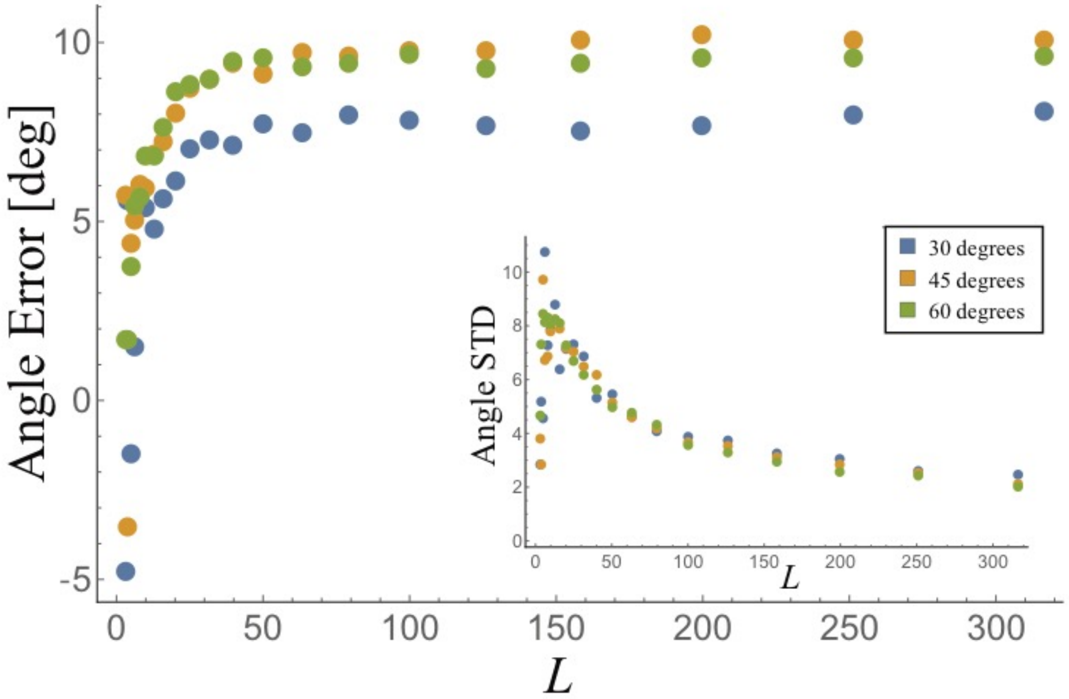

Figure S13: The dependence on base length and base angle of the dynamic model's angle estimates. The model captured the experimental results in the angle size estimation task. The model predictions showed increased overestimates of the missing angle size as triangle size increased (Base angles: 30 degrees – Blue, 45 degrees – Yellow, 60 degrees – Green). Inset, as base length increased, the variability in angle-size estimates decreased. Simulation parameters: integration time scale, τ=1, error-correction time scale, $\xi = 4$, speed $v_p = 1$, base length range, $B \in [10^{0.5}, 10^{2.5}]$, interior angles noise levels, $D = 0.1$, and stopping criterion threshold, $\epsilon = 0.2\ L$.

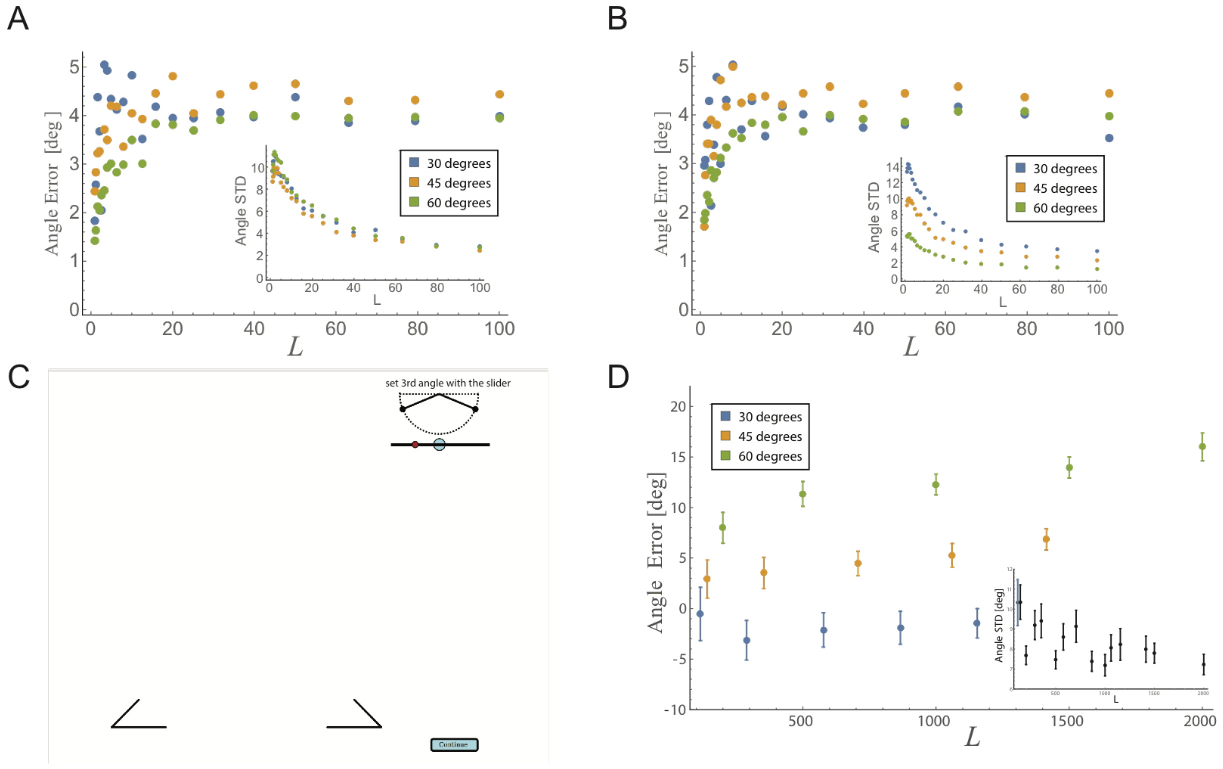

Figure S14: Participants estimate of missing angle size is predicted by the mathematical model. A,B) The model predicted that as side length increased, angle size overestimated errors increased (Base angles: 30 degrees – Blue, 45 degrees – Yellow, 60 degrees – Green). Inset, as side length increased, angle-size variability decreased. Shown are results for A) two separate angle assessments and B) an averaged location estimate with its corresponding missing angle **C)** Participants were shown two base corners angles of an isosceles incomplete triangle and were asked to move a slider to mark the missing corner angle size. **D)** Participants' angle sizes are overestimated with errors increasing as side length increased for 45 and 60 degrees. For 30 degrees, the curve of angle estimate errors did not show a clear trend (Base angles: 30 degrees – Blue, 45 degrees – Yellow, 60 degrees – Green). Inset, as side length increased, the variability in angle-size estimates decreased.

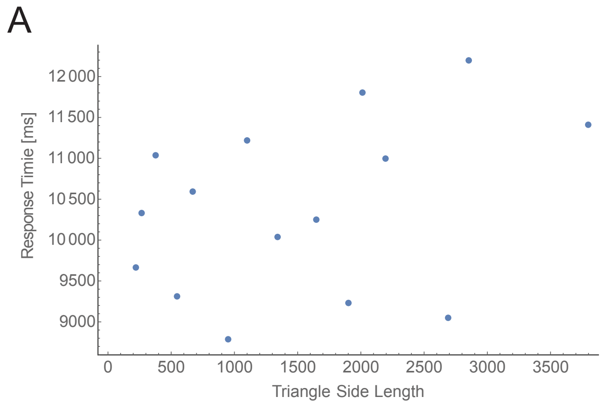

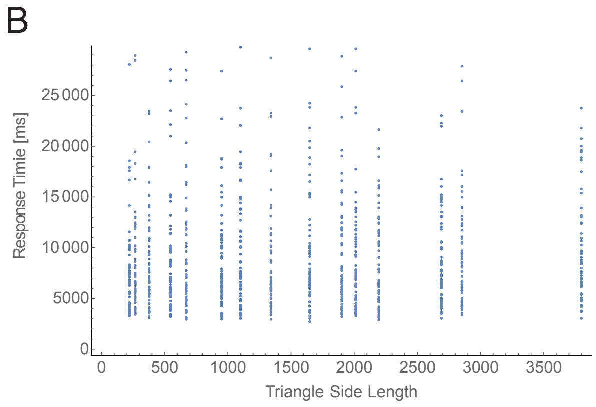

Figure S15: Response times correlate with triangle side length in the angle estimation task. A) We analyzed the response times of participants on the different triangles in the angle estimation task. We find that the mean response time per triangle size across all participants, response times shows a non-significant correlation with triangle's side length (Spearman correlation r=0.35, p-value=0.24). **B)** Similar analysis across all the response times shows a significant weak correlation with triangle's side length (Spearman correlation r=0.07, p-value<0.03). We note that in the angle estimation task, participants were using a slide bar to set the size of the missing angle.

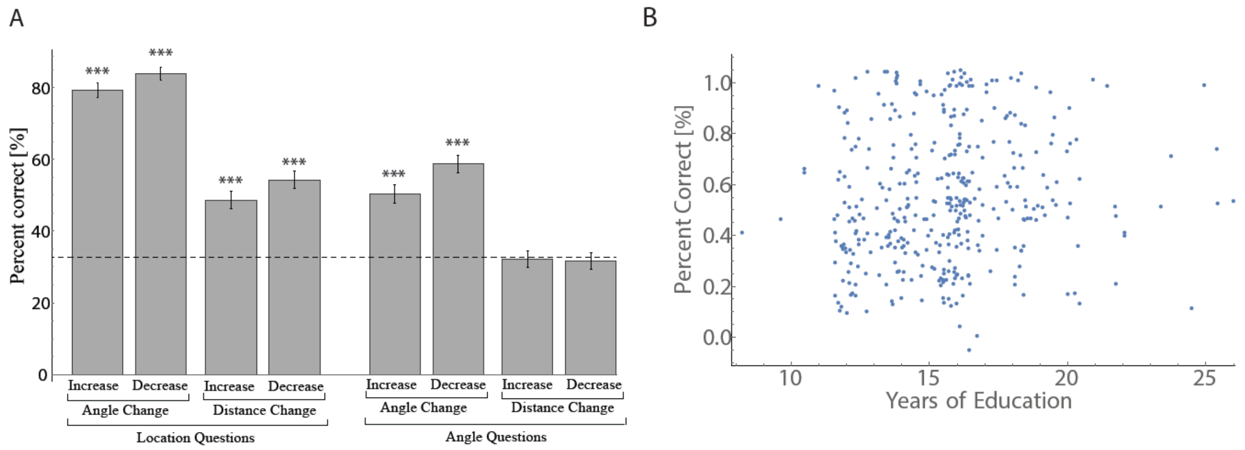

Figure S16: Participants education does not affect their accuracy. A) While participants succeeded in all questions probing the location of the triangle's missing vertex (angle change: Mean±STE=82±2%, distance change: Mean±STE=51±2%), they only succeeded in questions probing the angle size of that vertex after changes to the angle size (Mean±STE=55±2%) but not distance between the other two vertices (Mean±STE=32±2%). Dashed line is set at chance level (33%). *p<0.05, **p<0.01 (Bootstrapping 1000 times the data and compared to a Binomial distribution with B(n=407,p=1/3). **B)** We calculated the correlation between participants' percent correct and their years of education in the categorical geometric reasoning task. We randomly sampled the data 1000 times with returns and calculated the correlation in the data. We found no correlation between the percent of correct answers and participants' years of education. Median Spearman r=0.002, 95% CI=[0,0.28].

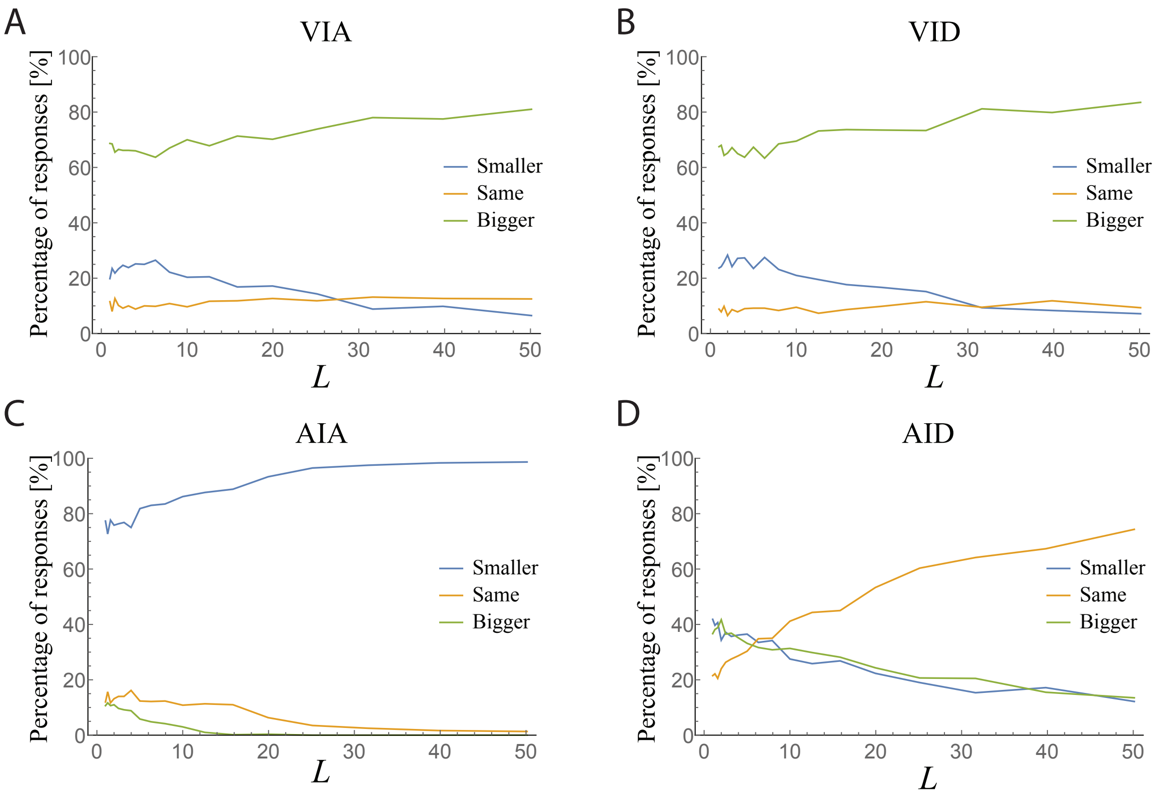

Figure S17: The dependence on initial triangle side-length of the model predictions for the categorical geometric reasoning task A) VIA condition **B)** VID condition **C)** AIA condition **D)** AID condition

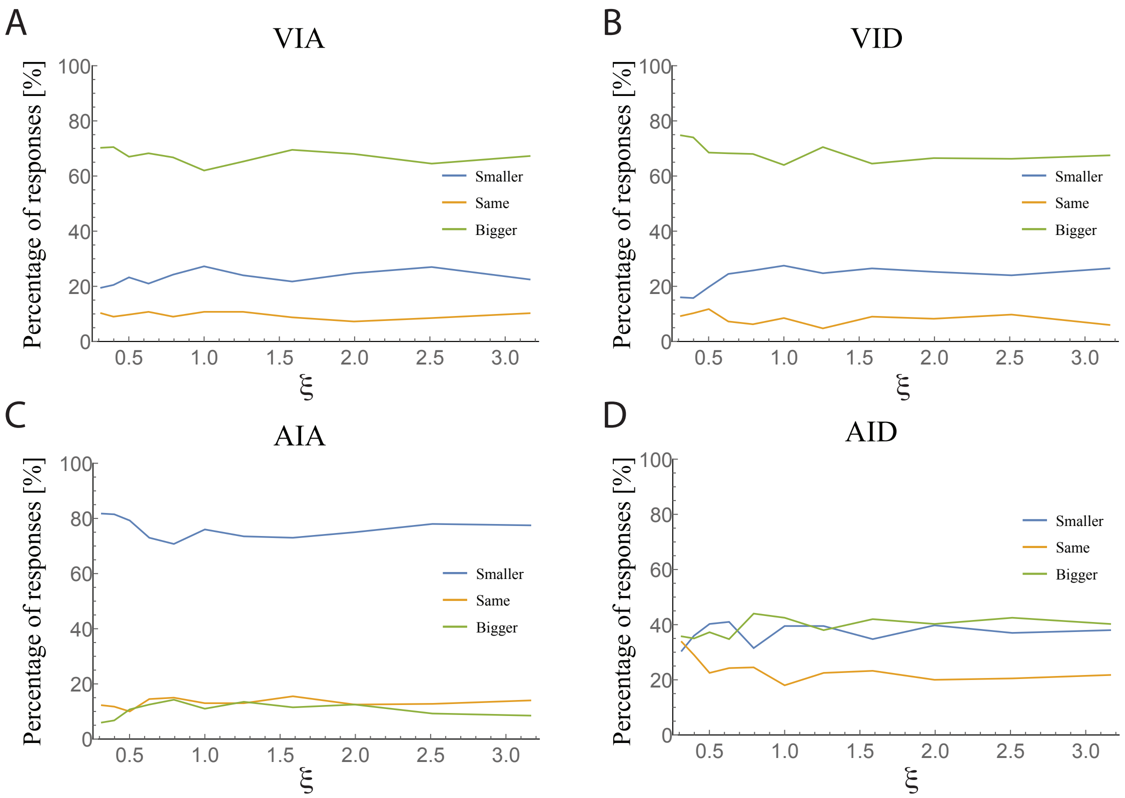

Figure S18: The dependence on correlation length, ξ, of the model's predictions for the categorical geometric reasoning task A) VIA condition **B)** VID condition **C)** AIA condition **D)** AID condition

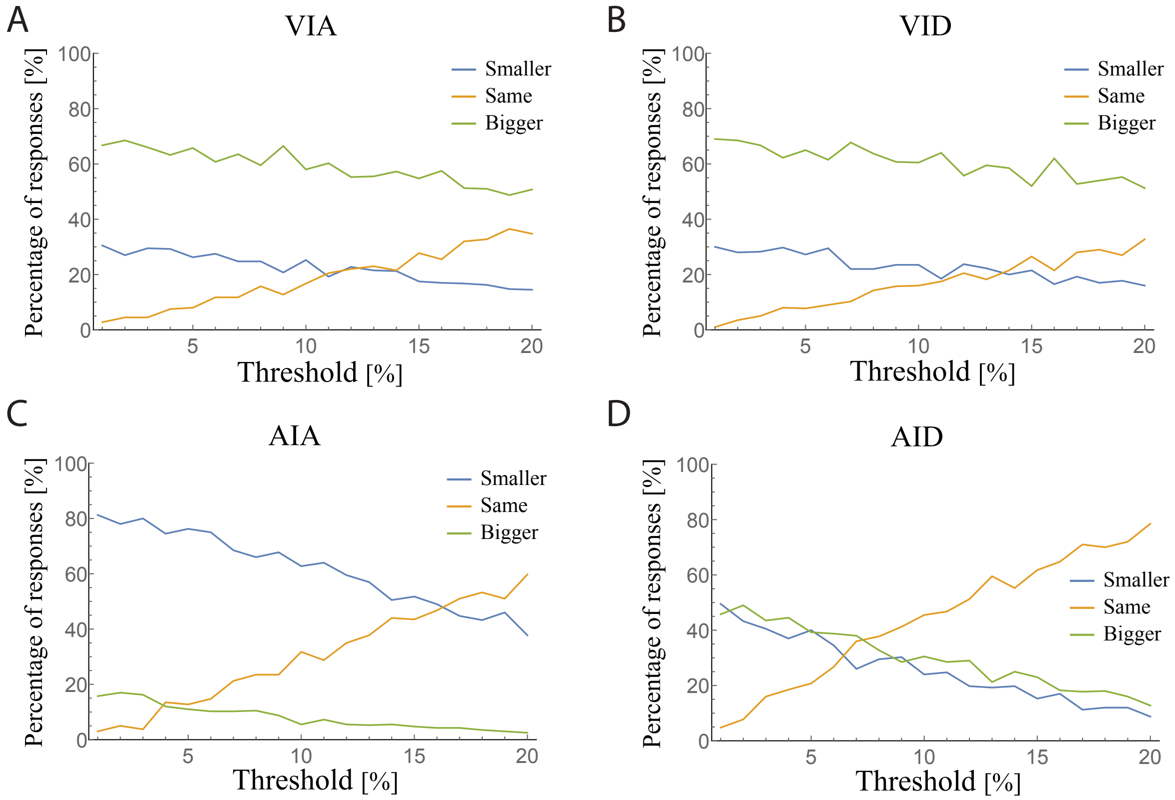

Figure S19: The dependence on threshold value of the model's predictions for the categorical geometric reasoning task A) VIA condition **B)** VID condition **C)** AIA condition **D)** AID condition

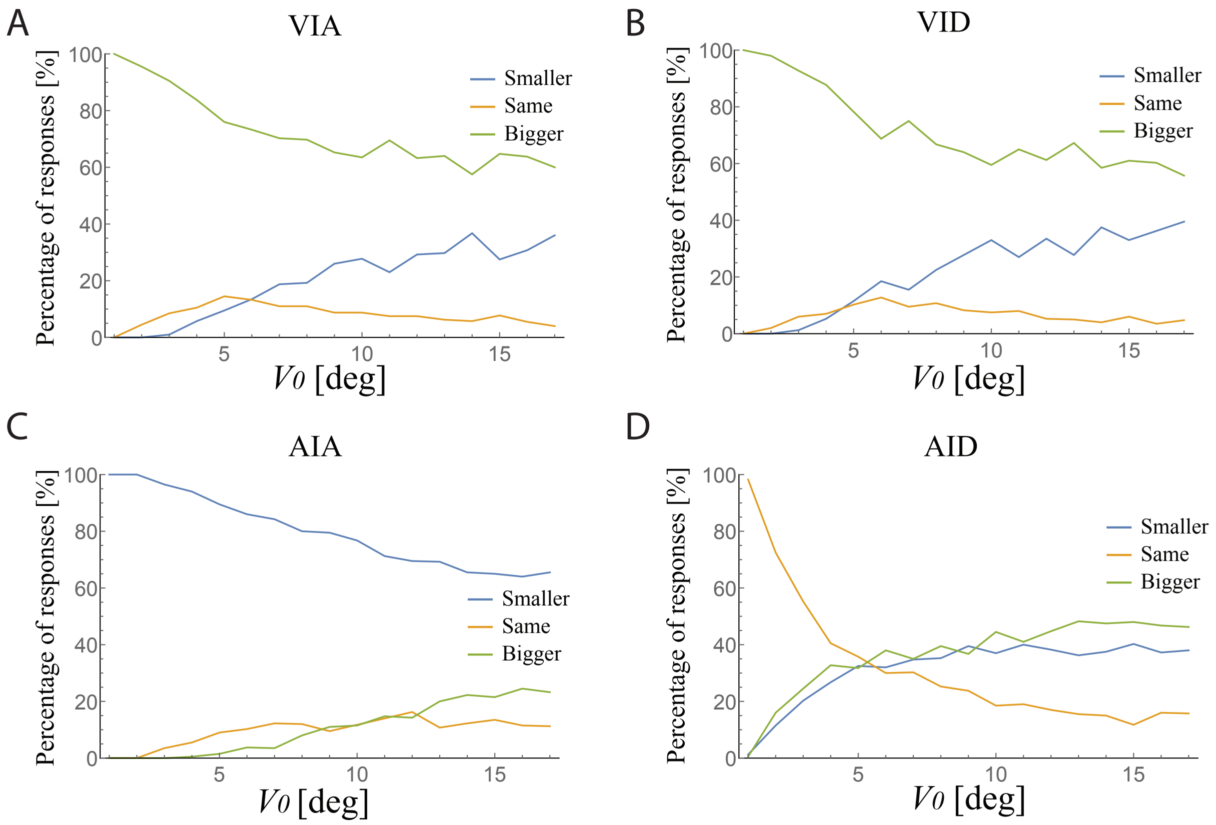

Figure S20: The dependence on the standard deviation of interior-angles estimates, V_0, of the model's predictions for the categorical geometric reasoning task A) VIA condition **B)** VID condition **C)** AIA condition **D)** AID condition

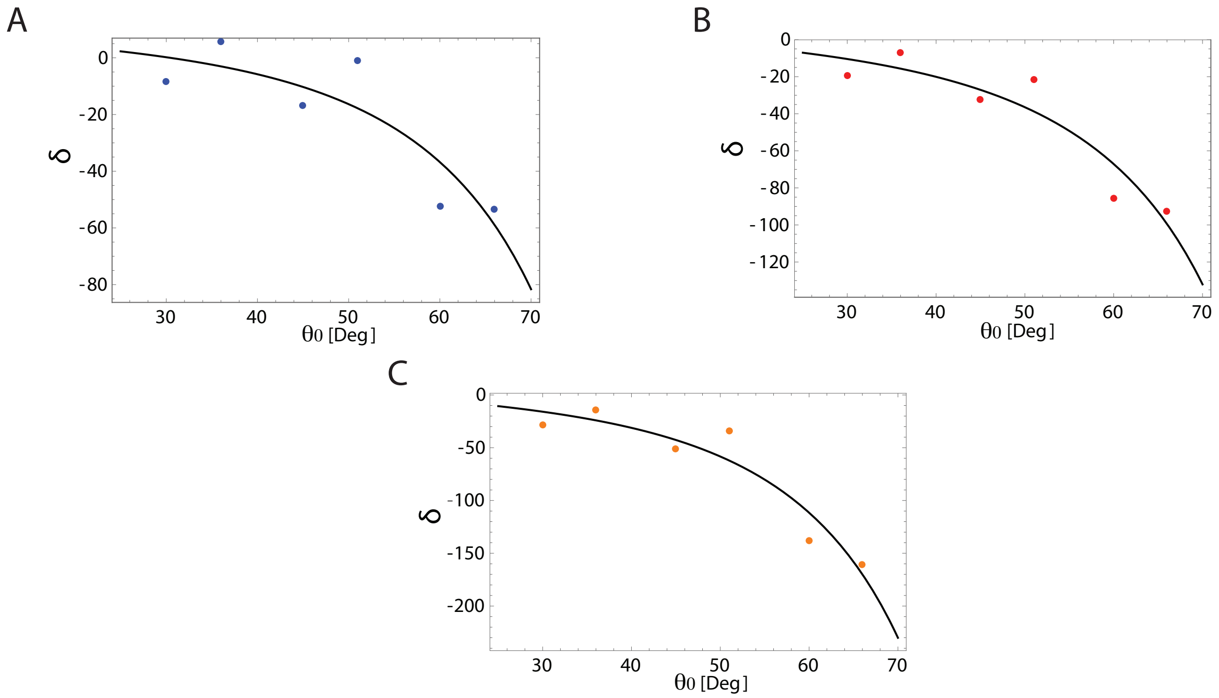

Figure S21: Observed bias in the y-coordinate follows the predicted scaling with base angle. In an online experiment, we asked participants (N=100) to mark the location of the missing corner of an incomplete triangle. A simple model of a noisy base-angle estimate captures the observed dependence between the errors in the vertical location estimates, δ, and base-angle sizes, θ_0. The model equation for the bias dependence the base-angle is: $\delta = -\frac{B}{2}\frac{2\,\sigma_0^2 \tan\theta_0}{2\,\sigma_0^2+\cos^2\theta_0}$, where B and σ_0 are fitting parameters. Shown are the localization task estimates of the vertical location for different base-angle sizes (30, 36, 45, 51, 60, and 66 degrees, see also Fig. S3). We note that while the model seems to fit well with the experimental data, the model's parameters showed a high variance in their estimated values. The model's parameters are (mean±ste): A) base length fold 0.5, B=130±250, $\sigma_0 = 0.3$±0.5. B) base length fold 0.75, B=130±60, $\sigma_0 = 0.6$±0.4 C) base length fold 1, B=240±120, $\sigma_0 = 0.5$±0.3.
